# Cell Cycle Kinetics and Sister Chromatid Exchange in Mosaic Turner Syndrome

**DOI:** 10.3390/life14070848

**Published:** 2024-07-05

**Authors:** Miriam Beatriz Goulart, Eduardo Vieira Neto, Daniela R. Ney Garcia, Marília Martins Guimarães, Isaías Soares de Paiva, Karina de Ferran, Nathalia Correia Krause dos Santos, Luciana Santos Barbosa, Amanda F. de Figueiredo, Maria Cecília Menks Ribeiro, Márcia Gonçalves Ribeiro

**Affiliations:** 1 Laboratory of Genetics, Institute of Childcare and Pediatrics Martagão Gesteira (IPPMG), Federal University of Rio de Janeiro (UFRJ), Rio de Janeiro 21941-912, RJ, Brazil; miriamgou@gmail.com (M.B.G.); daneygarcia07@gmail.com (D.R.N.G.); amandaffbr@gmail.com (A.F.d.F.); ceciliamenks@gmail.com (M.C.M.R.); marciaribeiro@medicina.ufrj.br (M.G.R.); 2 Genetic and Genomic Medicine Division, Department of Pediatrics, School of Medicine, University of Pittsburgh, Pittsburgh, PA 15224, USA; 3 Pediatric Endocrinology Service, IPPMG, UFRJ, Rio de Janeiro 21941-912, RJ, Brazil; marguima54@gmail.com (M.M.G.); karina.ferran@gmail.com (K.d.F.); nathaliakrausegenetica@gmail.com (N.C.K.d.S.); 4 Faculty of Medicine, University of Grande Rio (Unigranrio), Duque de Caxias 25071-202, RJ, Brazil; ispaivagen@gmail.com; 5 Faculty of Medicine, Serra dos Órgãos Educational Center (UNIFESO), Teresópolis 25964-004, RJ, Brazil; 6 NUMPEX-BIO Laboratory, Campus Duque de Caxias, UFRJ, Duque de Caxias 25240-005, RJ, Brazil; 7 Medical Genetics Service, IPPMG, UFRJ, Rio de Janeiro 21941-912, RJ, Brazil

**Keywords:** Turner syndrome, chromosomal mosaicism, cell lineages, proliferation index, sister chromatid exchange, chromosomal instability

## Abstract

Turner syndrome (TS) is caused by a complete or partial absence of an X or Y chromosome, including chromosomal mosaicism, affecting 1 in 2500 female live births. Sister chromatid exchange (SCE) is used as a sensitive indicator of spontaneous chromosome instability. Cells from mosaic patients constitute useful material for SCE evaluations as they grow under the influence of the same genetic background and endogenous and exogenous factors. We evaluated the proliferation dynamics and SCE frequencies of 45,X and 46,XN cells of 17 mosaic TS patients. In two participants, the 45,X cells exhibited a proliferative disadvantage in relation to 46,XN cells after 72 h of cultivation. The analysis of the mean proliferation index (PI) showed a trend for a significant difference between the 45,X and 46,X+der(X)/der(Y) cell lineages; however, there were no intra-individual differences. On the other hand, mean SCE frequencies showed that 46,X+der(X) had the highest mean value and 46,XX the lowest, with 45,X occupying an intermediate position among the lineages found in at least three participants; moreover, there were intra-individual differences in five patients. Although 46,X+der(X)/der(Y) cell lineages, found in more than 70% of participants, were the most unstable, they had a slightly higher mean PI than the 45,X cell lineages in younger (≤17 years) mosaic TS participants. This suggests that cells with a karyotype distinct from 45,X may increase with time in mosaic TS children and adolescents.

## 1. Introduction

The process of aneuploidy that occurs due to chromosome missegregation during meiosis is a major cause of birth defects, infertility, and spontaneous miscarriages [1]. Most constitutional aneuploidies cause embryonic lethality, the most notable exception in humans being trisomy 21, also known as Down syndrome (DS) [2].

Monosomies are relatively common events in early embryonic development, but survival beyond the fetal period is exceptional [3]. The embryonic and fetal survival of 45,X concepts, although extremely low (0.01%), can result in live births, which can be explained by the fact that it is a monosomy of sex chromosomes.

Turner syndrome is due to a partial or total loss of the second sex chromosome, resulting in the development of highly variable clinical features, affecting 1 in 2500 female live births [4,5]. It is a unique chromosome aneuploidy observed in newborns [4,5,6]. Monosomy of the X chromosome is the cytogenetic hallmark for TS [4], but a wide variety of other anomalies of the X or Y chromosome have been found, including chromosomal mosaicism, with whole or part of a sex chromosome [4,5,6]. At least 15–25% of TS patients have mosaicism [4,5].

It has been suggested that 45,X lineages have a prolonged cell cycle that can affect the differentiation of tissues and organs [7]. These preliminary data indicate that there could be an association between a prolonged cell cycle and the phenotypic features in TS [3].

TS phenotype is a result not only of the genomic imbalance from deleted genes—especially those that escape X inactivation or are in the pseudoautosomal region—but also from epigenetic influences, as X-chromosome and autosomal DNA-methylation and gene expression profiles from TS cells differ from normal female 46,XX cells [8,9,10]. Nonetheless, chromosome instability (CIN) could also play a role in TS complex pathogenesis and clinical variability [8].

Several molecular mechanisms have been suggested as responsible for TS phenotype [9]. The complete or partial loss of a second X chromosome may change the cell processes and expression of various genes, including *SHOX* (short stature homeobox-containing gene, NM000451), located in pseudoautosomal region 1 (PAR1) of X and Y chromosomes, whose haploinsufficiency contributes to some clinical features of TS, like short stature and skeletal abnormalities [9]. However, specific aneuploidy effects that are unconnected to gene dosage, such as impaired meiosis caused by unpaired chromosomes, may also contribute to TS phenotype [2]. According to Álvarez-Nava and Soto-Quintana [3], most of the phenotypic features related to X monosomy in TS are due to the simultaneous modification of several gene products (proteins, ncRNA) that, although having small individual effects, could lead to cumulative actions bringing about a lengthening of the cell cycle, with consequences to TS embryos (embryonic lethality), but also to children and adults with TS, being connected to short stature, gonadal dysgenesis, osteopenia/osteoporosis, congenital heart diseases, neurologic deficits, sensorineural hearing loss, and impaired pancreatic β-cell function.

Chromosome instability (CIN) is characterized by a high rate of loss and gain of whole-chromosome segments per cell cycle, resulting in cell-to-cell variability [11,12]. It is a hallmark of tumorigenesis and is also common during early human embryogenesis [11,12]. CIN can be associated with monosomy/trisomy of a whole chromosome and other genomic and chromosomal variations [3].

A higher incidence of sister chromatid exchange (SCE) can be associated with CIN, as is seen in some cancers and congenital syndromes [13,14,15,16,17]. Therefore, SCE has been used as a sensitive indicator of spontaneous CIN [13,18,19,20,21]. Previous research has reported high SCE incidences in patients with Bloom syndrome, Werner syndrome, and various types of neoplasia [13,14,21]. However, the genetic basis of high SCE incidences in these syndromes and neoplasias are pathogenic variants in DNA helicase genes (BLM in Bloom syndrome and WRN in Werner syndrome) that maintain genomic integrity and stability, having no similarities to the imbalances seen in constitutional aneuploidies.

SCE represents the interchange of DNA between replication products at apparently homologous loci and, at low levels, constitutes a normal occurrence. Although the molecular basis for SCE formation is not completely understood, it presumably involves DNA damage and repair [22,23].

On the other hand, there are few reports comparing cell cycle kinetics and SCE frequencies in mosaic TS patients until the present date. According to Melaragno et al. [19], mosaic patients are useful for SCE evaluation because the different cell lineages grow under the influence of the same exogenous and endogenous factors. Previously, Bortolai and Melaragno [24] analyzed one 45,X/46,XX patient in a small study series by SCE. The “mosaic” study design approach not only removes the confounding effects of interindividual differences due to total genetic makeup but also controls for the effects of environmental influences since both cell lines in individuals with mosaicism share identical exposure histories [25].

The main aim of the present cross-sectional study was to analyze the proliferation dynamics and SCE frequencies among 45,X and 46,XN lineages from a moderately large cohort of mosaic TS participants.

## 2. Materials and Methods

A total of 17 mosaic Turner syndrome (TS) participants were included in the present study from a cohort of circa 124 TS females diagnosed by the Cytogenetic Laboratory of Institute of Pediatrics and Childcare Martagão Gesteira (IPPMG) and followed by the Endocrinology Department of Clementino Fraga Filho University Hospital (HUCFF), Federal University of Rio de Janeiro (UFRJ), Brazil, from 1989 to 2022. The inclusion criteria were as follows: mosaic Turner syndrome subjects, ≤40 years of age, with two cell lines—a 45,X cell line co-occurring with a second cell line having whole X or Y chromosomes or X or Y structural abnormalities in at least 4% of the lymphocytes. A total of 17 mosaic TS participants were included: 13 with X or Y structural abnormalities and 4 with whole X or Y chromosomes. The study was approved by the Ethical Review Board of IPPMG, UFRJ (Ethical Review Board protocol n. 2.768.383). All participants or their parents gave their written consent.

Peripheral blood cultures were performed using the standard protocols modified from Moorhead et al. [26]. Peripheral blood from each participant was added to 5 mL of RPMI 1640 medium (Gibco, Grand Island, NY, USA), supplemented with 20% fetal bovine serum (Cultilab, Campinas, SP, Brazil) and phytohemagglutinin P (Cultilab).

The cultures were incubated for 48, 72, and 96 h. The cultures were harvested by standard procedures after one hour in MAS (Mitotic Arresting Solution, Genial Genetics, Chester, UK), treated with a hypotonic solution (0.075 M KCl), and fixed in Carnoy’s fixative, 3:1 methanol/glacial acetic acid (*v*/*v*). Cytological preparation and slide mounting were performed according to routine methods.

For bromodeoxyuridine (BrdU) incorporation, 50 µg/mL of BrdU (Sigma-Aldrich, Barueri, SP, Brazil) was added at the beginning of the cultures, which were incubated for 72 h. Differentiation staining was performed using a modified standard technique from previous studies [27,28].

The proliferation index (PI) after the BrdU labeling was conducted by scoring fifty metaphases from each cell lineage. A total of 100 metaphase cells (50 metaphases from 45,X and 50 from 46,XN cell lineages) from each participant were scored for the determination of the PI, calculated in 72-h cultures, according to the following equation: PI = (M1 + 2M2 + 3M3)/N, where M1, M2, and M3 represent the number of metaphases undergoing the first, second, and third divisions, and N is the total number of cells scored.

The frequencies of the 45,X and 46,XN lineages and evaluation of the in vitro selection were performed in 48-, 72-, and 96-h cultures, counting a total of 100 metaphases (50 cells from the 45,X lineage and 50 cells from the 46,XN lineage).

To appraise the chromosomal instability, sister chromatid exchange (SCE) frequencies among the 45,X and 46,XN lineages were compared. Twenty well-spread second-division metaphases from each cell lineage were analyzed, and the amount of SCE was computed for each cell.

The frequencies of SCE were compared between the 45,X and 46,XN lineages in an intra-individual analysis, comparing the lineages of the same individual participant, and in an intergroup analysis, considering the pool of monosomic and disomic cells in the group of participants.

Fluorescence in situ hybridization (FISH) was performed using the following commercial probes specific for chromosomes X and Y, according to manufacturers’ instructions: DXZ1/DYZ1 (centromeric regions, Xp11.1-q11.1 and Yq12; Vysis/Abbott, Abbott Park, IL, USA, or Zytovision, Bremerhaven, Germany), whole-chromosome painting (wcp) for X and Y (Cytocell, Milton, Cambridge, UK), SHOX (Xp22.33 and Yp11.3; Cytocell, Milton, Cambridge, UK), XIST (Xq13.2; Vysis/Abbott, Abbott Park, IL, USA, or Cytocell, Milton, Cambridge, UK), DYZ3 (centromeric region, Yp11.1-q11.1; Zytovision, Bremerhaven, Germany), ENXY (pericentromeric regions, Xq11.1 and Yq11.22.1; Lexel, Buenos Aires, Argentina), and SRY (Yp11.31; Vysis/Abbott, Abbott Park, IL, USA). All patients harboring marker chromosomes were screened with specific X/Y FISH probes.

For the statistical analysis, the Tukey’s and Šídák multiple comparisons tests were applied to compare the intergroup frequencies of lineages 45,X and 46,XX/46,XY—lineages with a second normal X or Y chromosome—and 46,X+der(X)/der(Y)—lineages with a second marker chromosome derived from X or Y, including the ring X chromosome and Y-derived isochromosome. Tukey’s test was used when considering different cultivation times of the same lineage, and Šídák for comparing both lineages of each participant at different cultivation times. The χ^2^ test or Fisher’s exact test was used to compare the frequencies of the 45,X and 46,XN lineages after different cultivation times in the same participant (intra-individual analysis). The two-tailed unpaired *t*-test was used for the intra-individual comparisons of the SCE frequencies of the 45,X and 46,XN lineages, while the χ^2^ test or Fisher’s exact test was used for the PI. One-way ANOVA with Dunnett’s post hoc multiple comparison test was used for the comparisons of the SCE frequencies between 45,X and several 46,XN karyotype groups. The means for the PI and SCE frequencies of lineages 45,X, 46,XX/46,XY, and 46,X+der(X)/der(Y) were compared by a two-tailed paired-t test. Pearson’s correlation coefficient (r) and linear regression were employed to measure the association of the PI and of the SCE frequencies between the 45,X, 46,XX/46,XY, and 46,X+der(X)/der(Y) lineages. Statistical analyses were performed using Prism version 10.2.3 for Mac (GraphPad Software, Boston, MA, USA) and Stata/SE 12.1 for Mac (Stata Corp, College Station, TX, USA) statistics software.

**Figure 1:**
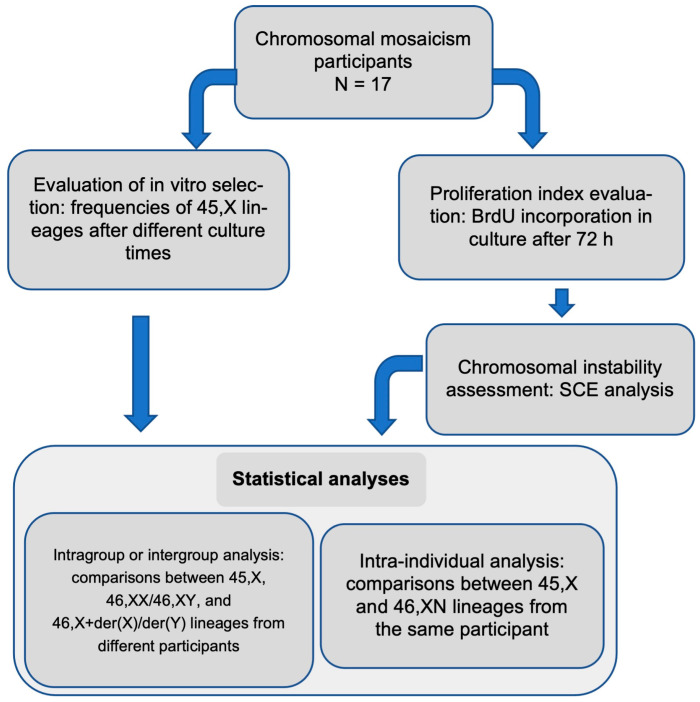
Flowchart showing the methods and statistical analyses used in this study.

The workflow below summarizes the methods employed in this study (Figure 1):

## 3. Results

The participants had a diagnosis of TS verified by karyotype and were selected from the medical records of the Laboratory of Genetics of IPPMG and the Pediatric Endocrinology Service, spanning more than 30 years of healthcare. From this cohort of circa 124 participants, 27 were included by convenience sampling and agreed to participate in a larger study constituting the PhD thesis of one of the authors (MBG). Ten participants were excluded from this specific study, as they presented exclusively with 45,X monosomy cell lineages. Therefore, a subset of 17 participants with mosaic TS comprised the sample of the present study. Their age ranged from 1.7 to 42 years (Table 1). Their phenotypes and other clinical characteristics are presented in Table S1.

Karyotype was confirmed using a standard cytogenetic technique and GTG-banding for the chromosomal analysis of participants 1, 2, 9, and 15, which showed besides 45,X, typical 46,XX (1, 2, 15) or 46,XY (9) lineages (Figures S1, S2, S9 and S15). Anomalous chromosomes found in 13 participants were characterized by FISH (Figures S3–S8, S10–S14, S16 and S17).

The FISH analysis showed that marker chromosomes were derived from the X chromosome in participants 5–8, 10–13, and 16 (Figures S5–S8, S10–S13 and S16). In two participants (4 and 14, Figures S4 and S14), the marker chromosomes were derived from the Y chromosome. A dicentric ring X chromosome was present in one participant (3, Figure S3), and another participant (17) presented an isochromosome of the short arm of the Y chromosome (Figure S17).

One copy of XIST (Xq13.2) was present in the marker chromosomes of six participants (5, 6, 10, 11, 13, and 16; Figures S5, S6, S10, S11, S13 and S16) and two copies in two participants, three within a dicentric ring chromosome (Figure S3), and twelve within a marker chromosome (Figure S12), both derived from X chromosome. In one participant (7, Figure S7), the XIST gene was not detected by hybridization on the marker chromosome.

The marker chromosome of participant 4 was positive for Y whole-chromosome painting and presented two copies of DYZ3, SRY, and SHOX, suggesting that it was a marker chromosome derived from the Y chromosome (Figure S4).

On the other hand, the marker chromosome of participant 14 presented two copies of ENY, DYZ3, and SRY, suggesting that it was an isodicentric marker chromosome derived from the Y chromosome (Figure S14). Participant 17 presented an isochromosome for the short arm of the Y chromosome with one copy of DYZ3 and two copies of ENY, SRY, and SHOX (Figure S17).

A catalog of loci on the X and Y chromosomes used to characterize anomalous chromosomes by FISH analyses is shown in Figure S18. A catalog of the marker chromosomes found in the participants is also shown in Figure S18.

### 3.1. In Vitro Selection

The occurrence of in vitro cell selection was verified by the amount of cells of the 45,X and 46,XN lineages in cultures after 48, 72, and 96 h of incubation. No significant effect of incubation time was found in the proportion of the two lineages for most participants, with two exceptions as follows:

- Participant 3: there was a decrease in the number of metaphases in the 45,X lineage and a simultaneous increase in the number of metaphases of lineage 46,X,+r(X) over the period (*p* = 0.0274).
- Participant 6: there was a decrease in the number of metaphases in the 45,X lineage and an increase in the number of metaphases in the 46,X,+mar(X) lineage when comparing the 48 h and 72 h incubations (*p* = 0.0234).

The frequencies of the 45,X and 46,XN lineages in the cultures incubated for 48, 72, and 96 h are shown in Figure S19.

**Figure 2:**
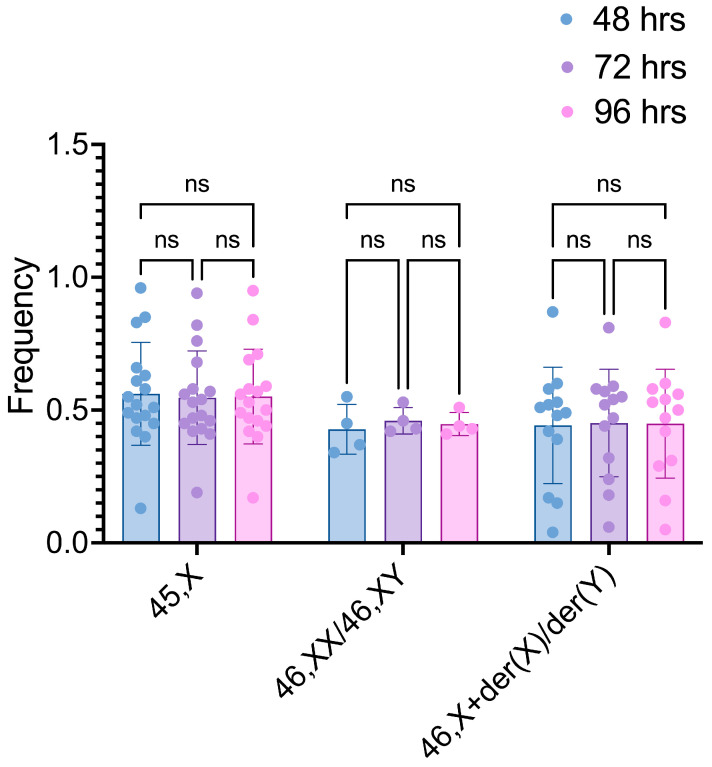
Frequencies of 45,X, 46,XX/46,XY, and 46,X+der(X)/der(Y) cell lineages after different culture times. Comparisons were conducted within each cell lineage. ns: non-significant.

The intergroup comparisons of the frequencies of the 45,X, 46,XX/46,XY, and 46,X+der(X)/der(Y) cell lineages after different cultivation times revealed no significant difference within the three lineages when 48 h vs. 72 h, 48 h vs. 96 h, and 72 h vs. 96 h were compared (45,X *p*-values = 0.6625, 0.8490, and 0.8803, respectively; 46,XX/46,XY *p*-values = 0.6412, 0.8429, and 0.5635, respectively; 46,X+der(X)/der(Y) *p*-values = 0.8887, 0.9471, and 0.9812) (Figure 2).

**Figure 3:**
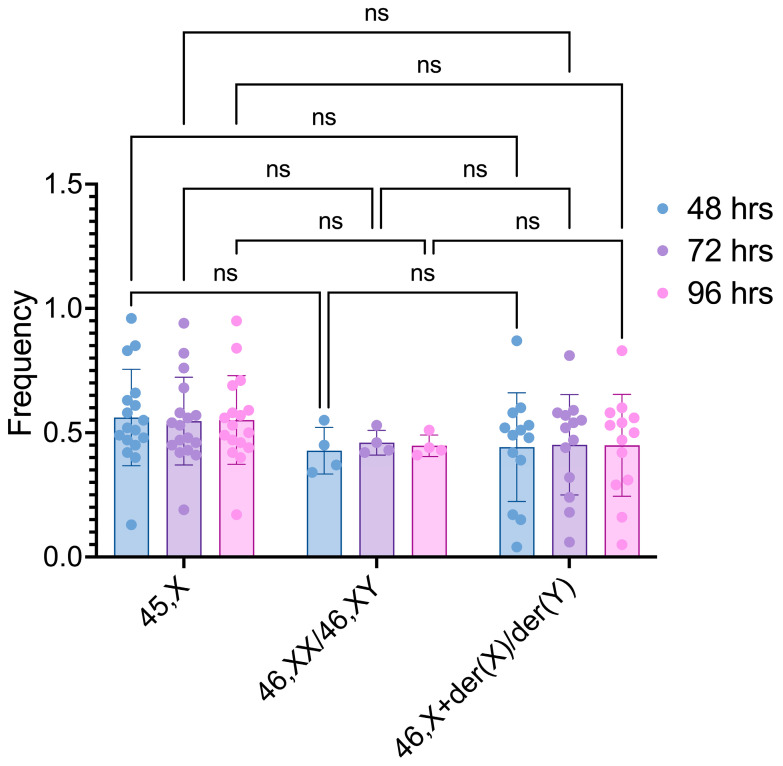
Frequencies of 45,X, 46,XX/46,XY, and 46,X+der(X)/der(Y) cell lineages after different culture times. Comparisons were conducted between cell lineages. ns: non-significant.

The comparisons between the three cell lineages after different cultivation times also did not detect any statistically significant differences (Figure 3).

### 3.2. Proliferation Index (PI)

**Figure 4:**
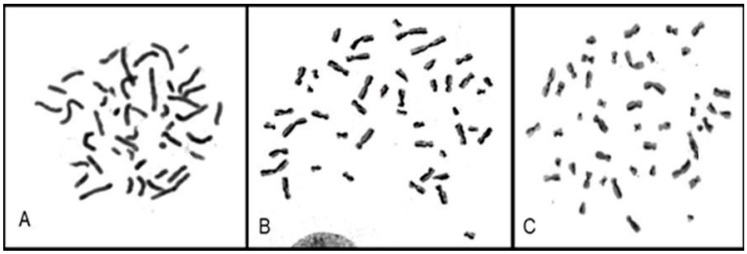
Characterization of cell division through the marking pattern of the chromatids in late prophase and early metaphase, which appear pale after substituting BrdU for thymidine on DNA strands. Both chromatids of cells in the first division are dark (**A**, participant 3); cells in the second division have one pale and one dark chromatid (**B**, participant 9), and 3/4 of the chromatids of cells in the third division appear pale (**C**, participant 3).

The proliferation index (PI) of each cell lineage was calculated individually by evaluating the number of cells in the first, second, and third cell divisions, according to the BrdU incorporation pattern (Figure 4).

**Figure 5:**
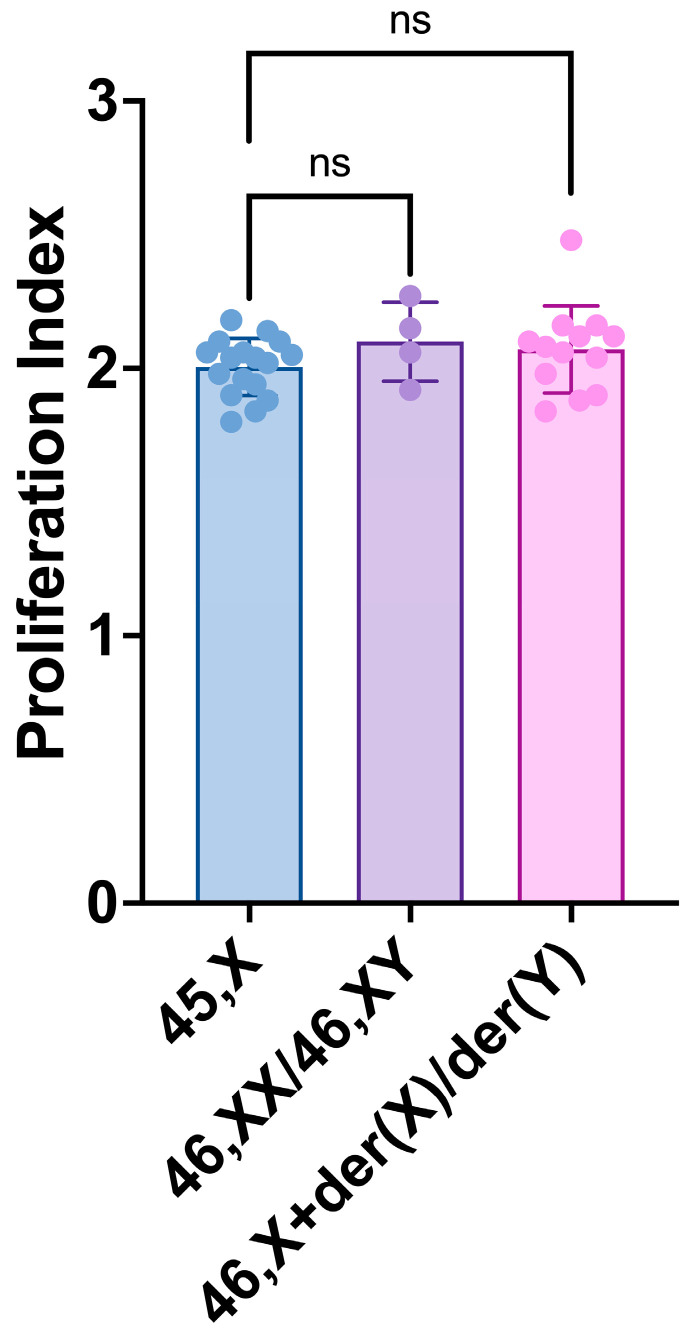
Proliferation index (PI) of 45,X, 46,XX/46,XY and 46,X+der(X)/der(Y) cell lineages. Scatter plots with bars show means with SD. Comparisons conducted between cell lineages’ means by paired *t*-tests: *p*-values: 45,X vs. 46,XX/46,XY = 0.4158, 45,X vs. 46,X+der(X)/der(Y) = 0.0610. The dots have the color of their parent bars (blue, purple, and pink) and represent the individual values of each category. There is a trend for a significant difference between the means of 45,X and 46,X+der(X)/der(Y) cell lineages. ns: non-significant.

The intra-individual comparisons of the PI between the 45,X and 46,XN lineages showed no significant differences (*p* > 0.5, χ^2^ test) in the participants with Turner syndrome and chromosomal mosaicism included in this study (Table 2). The intergroup comparison of the PI means of the 45,X cell lineage, considered as a reference, and the 46,XX/46,XY and 46,X+der(X)/der(Y) cell lineages showed no significant differences between 45,X and both 46,XN lineages (Figure 5). However, there was a trend (*p* = 0.061) for a significant difference between the 45,X and 46,X+der(X)/der(Y) cell lineages’ PI means.

There was a strong positive correlation between the PI of 45,X and 46,X+der(X)/der(Y) cell lineages (r = 0.6569, *p*-value = 0.0147). Conversely, there was no significant correlation between the PI of 45,X and 46,XX/46,XY cell lineages (r = 0.4284, *p*-value = 0.5716). Indeed, the correlation between the PIs of the 45,X and 46,X+der(X)/der(Y) cell lineages was confirmed by linear regression (*p*-value = 0.0147).

### 3.3. Sister Chromatid Exchange (SCE) Frequency

Chromosomal instability was assessed by calculating the SCE frequencies in 20 s-division metaphases of each cell lineage in 72-h cultures after BrdU labeling. The pictures of the second-division metaphases of the 17 participants, highlighting SCEs, along with first and third-division metaphases, which are employed in the PI calculation, are depicted in Supplementary Figures S20–S22.

The intra-individual comparisons of the SCE frequencies using the unpaired *t*-test showed a significant difference between the two cell lineages in five participants (2, 3, 4, 7, and 12) (Table 2).

In three participants (3, 7, and 12), the SCE frequencies were higher in the 46,XN lineage compared to the 45,X lineage. These 46,XN lineages presented anomalous chromosomes, where two participants had a marker chromosome derived from the X chromosome (46,X,+mar(X)), and the third had a dicentric ring chromosome derived from the X chromosome (46,Xr(X)).

**Figure 6:**
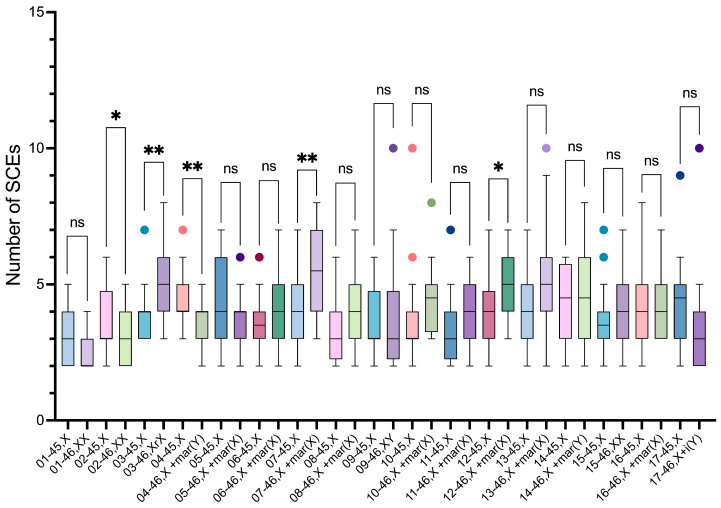
Intra-individual comparisons of SCE frequencies of 45,X and 46,XN lineages from the 17 participants. Box-and-whisker plots of the two cell lineages of each participant in contrasting different colors with outliers plotted as individual points beyond the whiskers on the boxplots, of the same color of parent box. There were significant differences between the lineages of participants 2, 3, 4, 7, and 12. ns, non-significant (*p* > 0.05), * *p* ≤ 0.05, ** *p* ≤ 0.01.

In two participants, the SCE frequencies were higher in the 45,X lineage. In participant 2, it was higher in relation to the 46,XX lineage, and in participant 4, it was higher in relation to the lineage with a marker chromosome derived from the Y chromosome (46,X+mar(Y)) (Figure 6).

**Figure 7:**
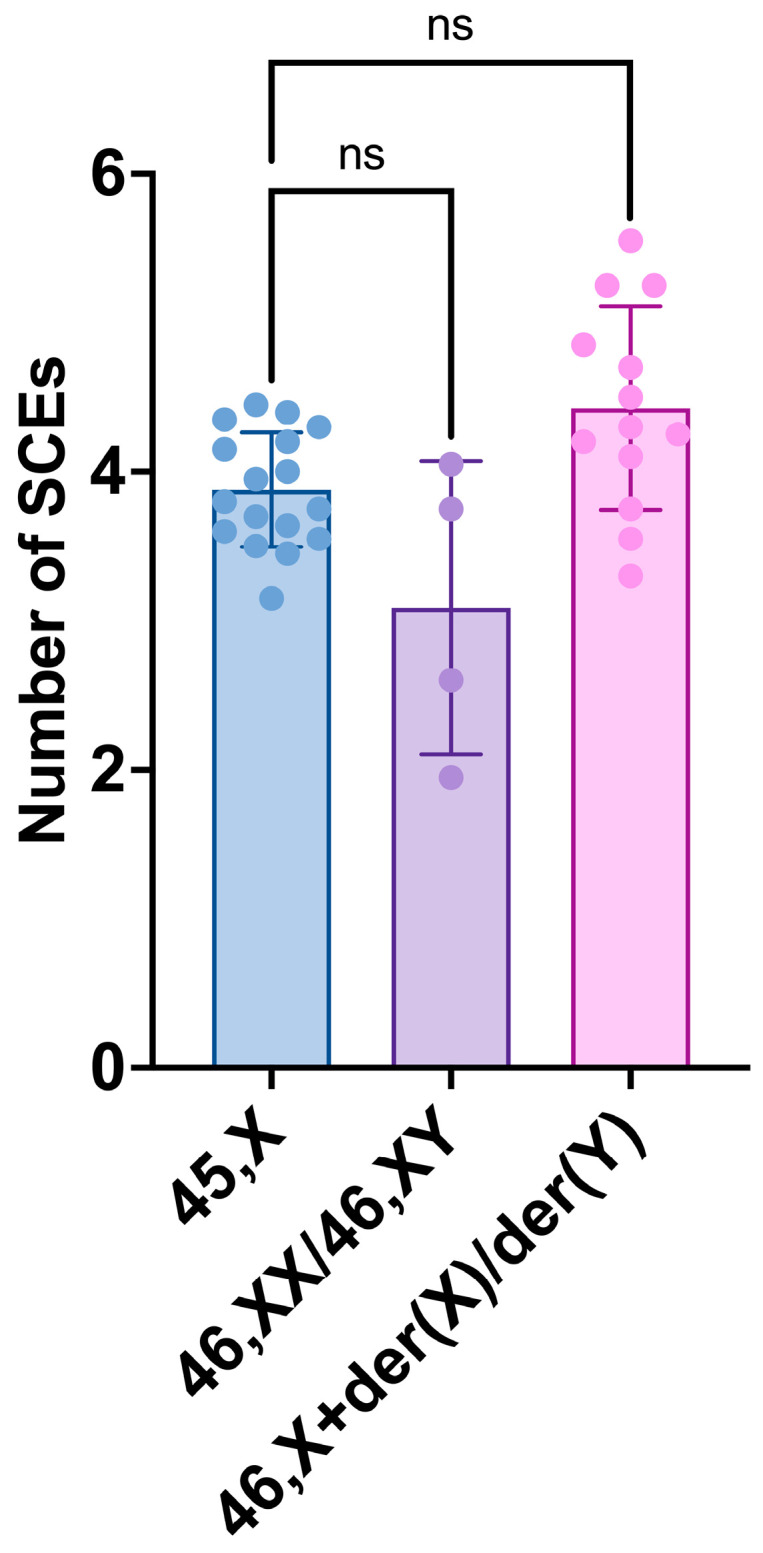
Sister chromatid exchange (SCE) frequencies of 45,X, 46,XX/46,XY, and 46,X+der(X)/der(Y) cell lineages. Scatter plots with bars show means with SD. The dots have the color of their parent bars (blue, purple, and pink) and represent the individual values of each category. Comparisons conducted between cell lineages’ means by paired *t*-tests: *p*-values—45,X vs. 46,XX/46,XY = 0.3967, 45,X vs. 46,X+der(X)/der(Y) = 0.0631. There is a trend for a significant difference between the means of 45,X and 46,X+der(X)/der(Y) cell lineages. ns: non-significant.

Regarding the intergroup comparisons of the SCE frequencies of the 45,X cell lineage, considered as a reference, and the 46,XX/46,XY and 46,X+der(X)/der(Y) cell lineages, there were no significant differences between 45,X and both 46,XN lineages (Figure 7). However, there was a trend (*p* = 0.0631) for a significant difference between 45,X and the 46,X+der(X)/der(Y) cell lineages’ SCE frequencies.

There were no significant correlations between the SCE frequency of the 45,X cell lineage, considered as a reference, and those of the 46,XX/46,XY (r = 0.4083, *p*-value = 0.5917) and 46,X+der(X)/der(Y) (r = −0.03076, *p*-value = 0.9205) cell lineages. Additionally, linear regression indicated that the relationship of the SCE frequencies of the three lineages was not statistically significant (*p*-values = 0.5917 and 0.9205, respectively).

**Figure 8:**
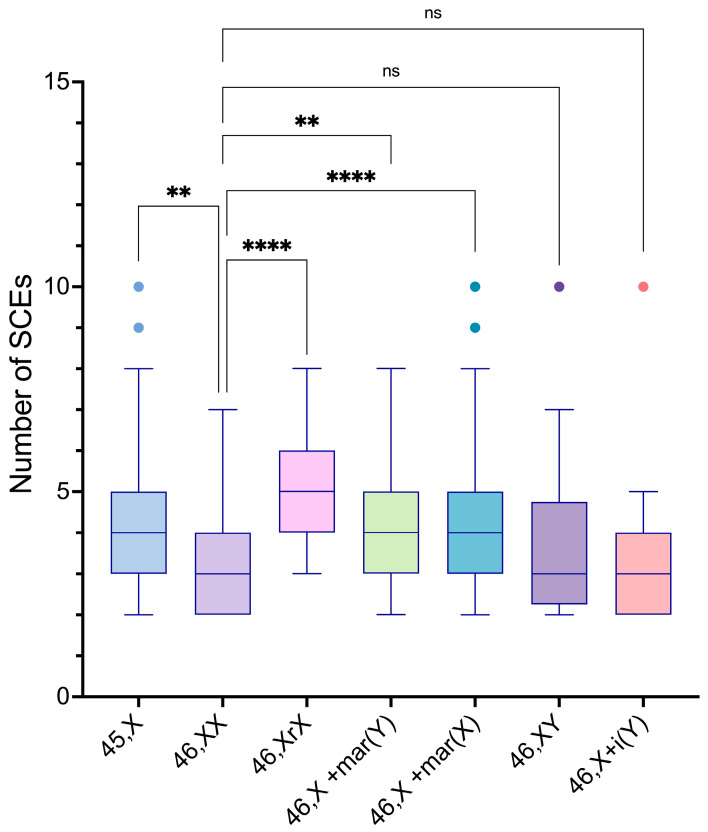
Intergroup comparisons of SCE frequency means between 46,XX lineage, considered as a reference group, and 45,X and the various 46,XN lineages found in the study participants. Box-and-whisker plots of the different cell lineages found in the participants represented in contrasting different colors with outliers plotted as individual points beyond the whiskers on the boxplots, of the same color of parent box. There were significant differences between 46,XX lineage, and 45,X, 46,Xr(X), 46,X+mar(Y), and 46,X+mar(X) lineages (*p*-values = 0.0155, <0.0001, 0.0328 and <0.0001, respectively). ns, non-significant (*p* > 0.05), ** *p* ≤ 0.01, **** *p* ≤ 0.0001.

The intergroup comparisons of the SCE frequencies among the 45,X and the distinct 46,XN lineages showed statistically significant differences between the 45,X lineage and the 46,XX, 46,X,r(X), and 46,X,+mar(X) lineages (*p*-values = 0.0155, 0.0007, and <0.0001, respectively); between the 46,XX lineage and the 46,Xr(X), 46,X,+mar(Y), and 46,X,+mar(X) lineages (*p*-values = <0.0001, 0.0328, and <0.0001, respectively); between the 46,X,r(X) lineage and the 46,XY and 46,X,i(Yp) lineages (*p*-values = 0.0198 and 0.0004, respectively); and the 46,X,+mar(X) lineage and 46,X,i(Yp) lineage (*p*-value = 0.0060) (Figure 8).

### 3.4. Age Effects on Proliferation Index (PI) and Sister Chromatid Exchange (SCE) Frequency

The 45,X and 46,X+der(X)/der(Y) cell lineages were divided into two age groups: 1.7–17 years, younger group, and 19–40 years, older group. The 46,XX/46,XY cell lineages and their corresponding 45,X cell lineages found in the same participants were excluded, as all four of them would be in the older group, 19–40 years, thus introducing biases to the analyses.

**Figure 9:**
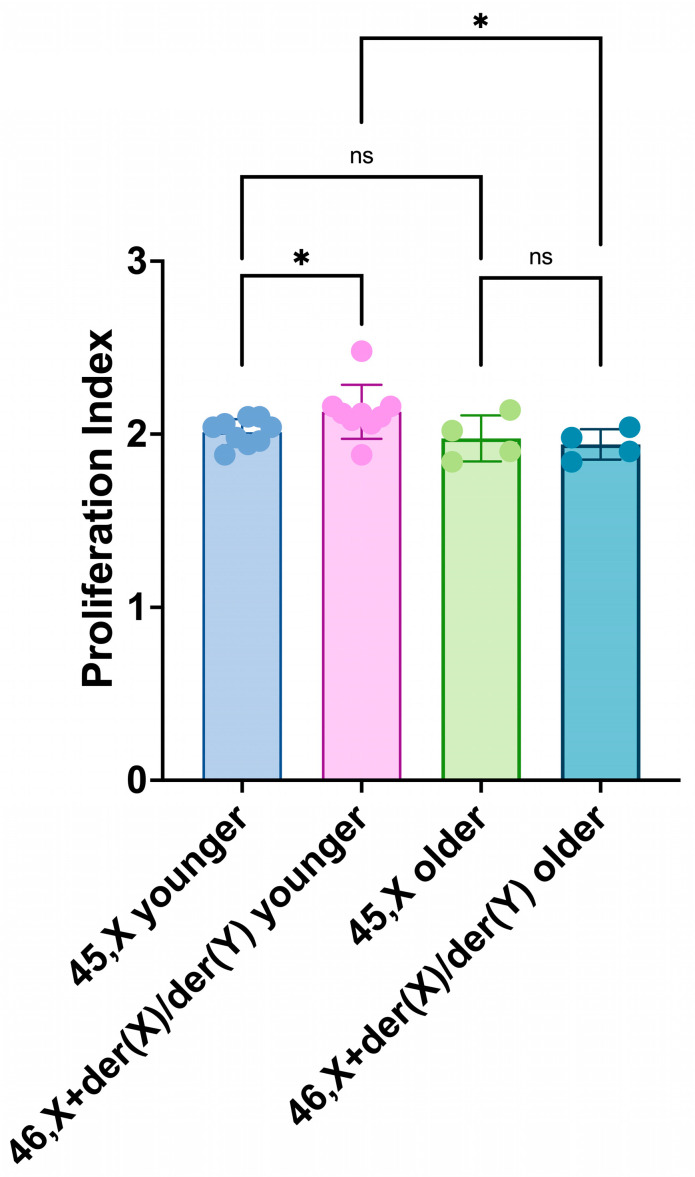
Proliferation index (PI) of 45,X and 46,X+der(X)/der(Y) cell lineages of younger (1.7–17 years) and older (19–40 years) groups. Scatter plots with bars show means with SD. The dots have the color of their parent bars and represent the individual PI values of each participant cell lineage; younger participants: blue and pink; older participants: lime and fern. Comparisons conducted between cell lineages’ means of the same group by paired *t*-tests, and of the opposite group by unpaired *t*-tests: *p*-values—younger group 45,X vs. 46,X+der(X)/der(Y) = 0.0148; older group 45,X vs. 46,X+der(X)/der(Y) = 0.3794; younger group 45,X vs. older group 45,X = 0.5390; younger group 46,X+der(X)/der(Y) vs. older group 46,X+der(X)/der(Y) = 0.0475. There is a slight but significant proliferative advantage of 46,X+der(X)/der(Y) of younger participants over 45,X cell lineages that seems to dissipate as they grow older. ns, non-significant (*p* > 0.05), * *p* ≤ 0.05.

The PI of the 46,X+der(X)/der(Y) cell lineages of the younger group was slightly but significantly higher than the 45,X of the same group and 46,X+der(X)/der(Y) cell lineages of the older group (Figure 9).

**Figure 10:**
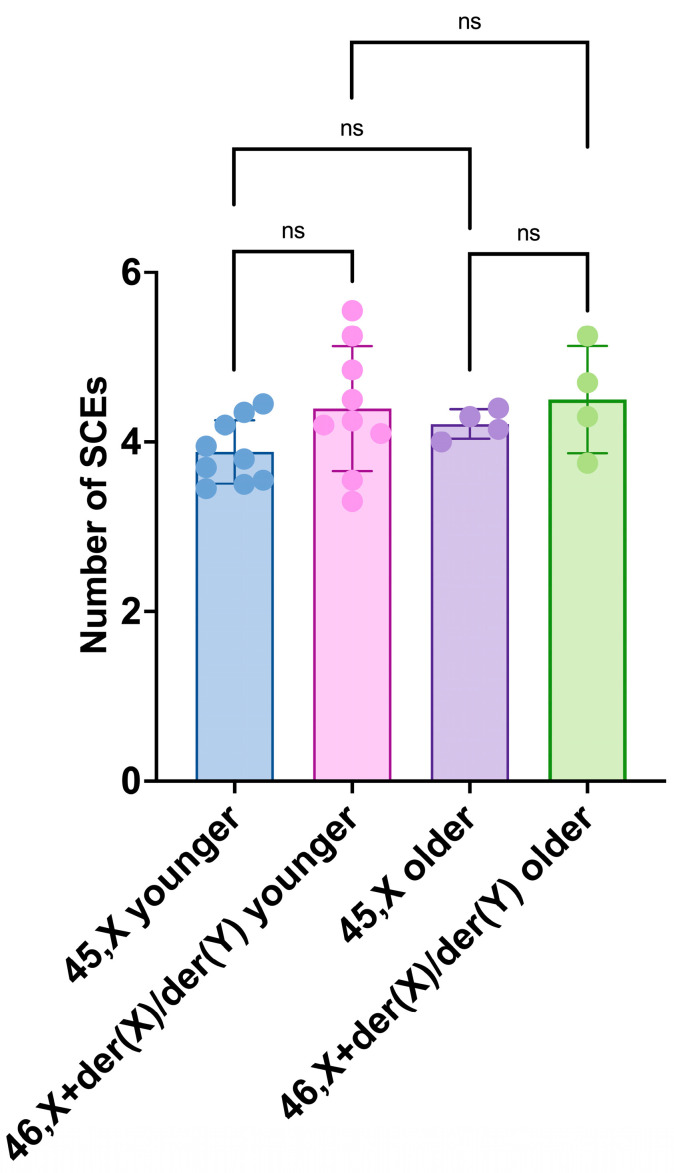
Sister chromatid exchange (SCE) frequencies of 45,X and 46,X+der(X)/der(Y) cell lineages of younger (1.7–17 years) and older (19–40 years) groups. Scatter plots with bars show means with SD. The dots have the color of their parent bars and represent the individual SCE values of each participant cell lineage; younger participants: blue and pink; older participants: purple and lime. Comparisons conducted between cell lineages’ means of the same group by paired *t*-tests, and of the opposite group by unpaired *t*-tests: *p*-values—younger group 45,X vs. 46,X+der(X)/der(Y) = 0.1205; older group 45,X vs. 46,X+der(X)/der(Y) = 0.3736; younger group 45,X vs. older group 45,X = 0.1285; younger group 46,X+der(X)/der(Y) vs. older group 46,X+der(X)/der(Y) = 0.8092. No significant differences were found in the intra- and intergroup comparisons. ns: non-significant.

The SCE frequencies of the 45,X and 46,X+der(X)/der(Y) cell lineages of the younger and older groups, compared intragroup by paired *t*-tests or intergroup by unpaired *t*-tests, did not show any significant differences (Figure 10).

## 4. Discussion

The present study evaluated the in vitro selection and PI and SCE frequencies of 17 mosaic TS participants. As far as we know, this is the largest cohort of mosaic TS individuals to be evaluated for these parameters. Chromosomal instability and lymphocyte proliferation have been more commonly evaluated in Down syndrome individuals, either by comparing the SCE frequencies of trisomic cell lineages with those of unaffected individuals or by comparing the SCE frequencies of normal cell lineages with trisomic ones in mosaic Down syndrome [25,29]. Recently, Rafferty et al. [25] compared the chromosomal instability of disomic and trisomic cell lineages in mosaic Down syndrome individuals. The authors observed a significantly higher spontaneous chromosomal instability in trisomic cells compared to isogenic disomic cells from mosaic Down syndrome individuals. In addition, chromosomal instability in trisomic cells increased with the age of the individuals. However, these findings are hardly extrapolated to our cohort of mosaic TS individuals, as the chromosomal imbalance is brought principally by 45,X monosomy. According to Álvarez-Nava and Lanes [8], not only is the loss of genes caused by chromosomal imbalance due to 45,X monosomy, responsible for the TS phenotype, but it is also caused by the epigenetic factors triggered by the absence of the second sex chromosome. However, epigenetic processes have been poorly studied in TS.

TS individuals who carry structurally abnormal chromosomes are a unique group, and they have provided opportunities to appraise the cellular consequences of chromosomal imbalance.

Evidence of in vitro selection was observed in only two of seventeen participants. Participant 3, displaying a dicentric ring X chromosome, and 6, displaying a marker chromosome derived from X chromosome, both showed a decrease in monosomic cells after increased cultivation times, suggesting that there was a selective disadvantage (in vitro) for the 45,X lineage in these participants. However, the intergroup comparisons of the frequencies of the 45,X, 46,XX/46,XY, and 46,X+der(X)/der(Y) cell lineages revealed no significant differences in the proportions of 45,X, 46,XX/46,XY, and 46,X+der(X)/der(Y) lineages after cultivation over time. Summarizing, the results from most of the participants in this study did not reveal differences in the 45,X, 46,XX/46,XY, and 46,X+der(X)/der(Y) cell lineages’ proportions in culture over time. However, differences were found eventually in some (12%) mosaic TS individuals.

Bortolai and Melaragno [24] also did not observe statistically significant differences between the proliferation of aneuploid and normal cells of a mosaic 45,X/46,XX TS patient after 48, 72, and 96 h cultivation times. We did not detect the in vitro selective disadvantage of the 45,X lineage in three 45,X/46XX and one 45,X/46XY participants in this study (Figure S19). It is possible that cultures for periods longer than 96 h are required to detect the in vitro selective disadvantage of 45,X cell lineages.

The PI means intra-individual comparisons showed no significant differences between the 45,X and 46,XN lineages. The intergroup comparison of the PI means of the 45,X and 46,XX/46,XY cell lineages also did not show a significant difference (paired *t*-test *p*-value = 0.4158), but there was a trend for a difference between the 45,X and 46,X+der(X)/der(Y) cell lineages (paired *t*-test *p*-value = 0.0610). When the participants were divided into two age groups, the proliferative advantage of 46,X+der(X)/der(Y) in relation to 45,X was very clear in the younger group (paired *t*-test *p*-value = 0.0148). Unfortunately, due to the small number of TS mosaic participants with the 46,XX/46,XY cell lineages, a similar analysis could not be conducted for those harboring normal diploid cell lines. A previous study with skin fibroblast cultures comparing the differences in the cell cycle of aneuploid cell lines (45,X) to cell lines with structural anomalies of the X chromosome and normal diploid cell lines, 46,XX and 46,XY, showed that the cell cycle of the 45,X cell lines were significantly longer compared to the euploid controls [3]. A possible explanation for this phenomenon would be a longer S phase, resulting in a prolonged inter-mitotic period in 45,X cells that could lead to a decrease in their quantity during organogenesis, which could contribute to important phenotypical features in TS [3,7].

It was a surprise to find an indication of a proliferative advantage of anomalous cell lineages, 46,X+der(X)/der(Y), over the 45,X cell lineages in the younger group. If this is confirmed in future studies, the proportion of anomalous diploid cells may increase at least in younger TS mosaic individuals with age, as has already been found in those with normal diploid cells [30]. This contrasts with the increase in aneuploid cells due to a loss of the X chromosome in unaffected women with age [31,32,33].

SCE are considered to be cytological manifestations of homologous recombination. They occur naturally as events associated with normal DNA replication, with estimates being 3–4 exchanges per cell per replication cycle [22,34,35,36]. In Bloom syndrome, a very high SCE frequency is found, well above that of cells from unaffected individuals or individuals with any other genetic disorder [37]. Nonetheless, Bloom syndrome is caused by pathogenic variants in *BLM*, a DNA helicase gene that is involved in genome replication, DNA repair, recombination, transcription, and telomere maintenance. Consequently, the mechanism involved in the high SCE frequency in Bloom syndrome cannot be extrapolated to constitutional aneuploidies.

Previous reports compared the cell cycle dynamics, cell selection, and chromosomal instability between abnormal and normal cell lineages in TS mosaic individuals, and no significant differences between the aneuploid and normal cell lines were observed [24,30]. SCE can also reflect genomic instability and may serve as a preclinical biomarker for the early detection of genomic instability [13,21,23,34,35,36,37]. Nonetheless, in this study, the amount of SCE in the participants was not remarkable, and the average was similar to the baseline.

The SCE frequencies were significantly different between the 45,X and 46,XN cell lineages in five participants. In three participants (3, 7, and 12), the SCE frequencies were higher in the lineage with a structurally anomalous sex chromosome compared to the 45,X lineage. In participant 2, the SCE frequency was higher in the 45,X lineage compared to the 46,XX lineage, and in participant 4, the SCE frequency of the 45,X lineage was higher than that of a lineage with a marker chromosome derived from the Y chromosome. More than 70% of the participants in our study presented a lineage with anomalous chromosomes, and only four were mosaics with a normal female (46,XX) or male (46,XY) cell lineage. Consequently, most of the intra-individual comparisons were between cell lineages with a certain amount of genomic instability.

Considering the cell lineages found in at least three participants, 46,XX cell lineages had the lowest SCE frequency (mean = 3.20), and the highest was found in 46,X+der(X) (mean = 4.53); 45,X cell lineages presented intermediary frequencies (mean = 3.88). These findings agree with Iqbal, Martin, and Simpson [38], who demonstrated significantly increased mean SCE frequencies in all fibroblast cell lines with abnormalities of the X chromosome: 45,X; 46,X,del(X)(q13); 46,X,del(X)(p11); and 46,X,i(Xq) compared to normal 46,XX cell lines. Our results with primary lymphocyte cultures confirm Iqbal, Martin, and Simpson [38], who employed fibroblast cell lines, and both studies may reproduce what is happening in mosaic TS tissues and organs.

The frequency of the Y chromosome in TS is variable [17,39], usually being found in 3 to 12% of individuals [9,39,40,41]. The presence of Y sequences in our study was 23.5% (4/17). Probably, this higher frequency in our study is due to the significant number of individuals with marker chromosomes. From the four participants with intact or anomalous chromosomes derived from the Y chromosome, only one showed significant differences in the SCE frequencies between the two lineages. Surprisingly, the 45,X lineage from this participant showed a higher SCE frequency than the lineage with a marker chromosome derived from Y chromosome cells.

As far as we know, the number of articles that have studied genomic instability in aneuploidies is limited. Yet, all data suggest that cells from Down syndrome, Edward syndrome, Turner syndrome, and Patau syndrome patients may be karyotypically less stable than cells from unaffected individuals [2]. Our cross-sectional study with a sample of mosaic TS selected by convenience, with a significant number of participants with marker chromosomes, showed some evidence that the 45,X cell lineage may have a selective disadvantage in younger individuals, although there was a significant variation concerning the differences in SCE frequencies between the 45,X, normal 46,XX, and anomalous 46,X+der(X) lineages. Therefore, the presence of chromosomal structural abnormalities, especially the presence of marker chromosomes, could have been responsible for the variation in the 46,XN lineage’s instability observed in our study.

## Figures and Tables

**Table 1:** Karyotypes of participants with Turner syndrome included in this study.

Participants	Karyotype Cytogenetics	Age
1	mos45,X[54]/46,XX[46]	27
2	mos45,X[58]/46,XX[42]	30
3	mos45,X[76]/46,X,+r[24].ish r(X)(DXZ1+,*XIST*++)	16
4	mos45,X[52]/46,X+mar[48].ish der(Y)(wcpY+,DYZ3++,*SHOX*++,*SRY*++)	11
5	mos45,X[58]/46,X,+mar[42].ish der(X)(DXZ1+,*XIST*+)	19
6	mos46,X,+mar[51]/45,X[49].ish der(X)(DXZ1+,*XIST*+)	11
7	mos45,X[94]/46,X,+mar[6].ish der(X)(DXZ1+,*XIST*-)	1.7
8	mos46,X,+mar[58]/45,X[42].ish der(X)(DXZ1+,*XIST*++)	12
9	mos46,XY[53]/45,X[47]	31
10	mos46,X,+mar[58]/45,X[42].ish der(X)(DXZ1+,*XIST*+)	15
11	mos45,X[51]/46,X,+mar[49].ish der(X)(DXZ1+,*XIST*+)	5
12	mos45,X[61]/46,X,+mar[39].ish der(X)(DXZ1+,*XIST*++)	17
13	mos45,X[82]/46,X,+mar[18].ish der(X)(ENX+,*XIST*+)	22
14	mos46,X,+mar[55]/45,X[45].ish der(Y)(ENY++,DYZ3++,*SRY*++)	23
15	mos45,X[57]/46,XX[43]	42
16	mos45,X[53]/46,X,+mar[47].ish der(X)(DXZ1+,*XIST*+)	19
17	mos46,X,i(Y)(p10)[81]/45,X[19].ish i(Y)(ENY+,DYZ3+,*SRY*++,*SHOX*++)	14

**Table 2:** Intra-individual comparisons of SCE frequencies and PIs of the two cell lineages of the participants.

Participants	Lineages	SCE Mean	*p*-Value	PI	χ^2^	*p*-Value
1	45,X/46,XX	3.15/2.6	0.0551	2.05/2.15	0.9387	0.6254
2	45,X/46,XX	3.65/2.95	0.0454 *	2.18/2.27	0.9859	0.6108
3	45,X/46,X,+r(X)	3.95/5.25	0.0046 **	2.06/2.16	1.016	0.6017
4	45,X/46,X,+mar(Y)	4.45/3.55	0.0046 **	2.10/2.10	0.1640	0.9213
5	45,X/46,X,+mar(X)	4.15/3.75	0.3583	2.02/1.98	0.7311	0.6938
6	45,X/46,X,+mar(X)	3.55/4.25	0.0779	2.04/2.12	0.4380	0.8033
7	45,X/46,X,+mar(X)	4.2/5.55	0.0100 **	2.10/2.10	0.1829	0.9126
8	45,X/46,X,+mar(X)	3.45/4.2	0.0714	1.88/1.88	0.1814	0.9133
9	45,X/46,XY	3.6/3.75	0.7805	2.06/1.92	2.123	0.3460
10	45,X/46,X,+mar(X)	3.7/4.5	0.1183	1.98/2.16	3.187	0.2032
11	45,X/46,X,+mar(X)	3.5/4.1	0.1544	2.04/2.12	0.4600	0.7945
12	45,X/46,X,+mar(X)	3.8/4.85	0.0105 *	1.94/2.08	1.432	0.4886
13	45,X/46,X+mar(X)	4.3/5.25	0.0745	1.84/1.90	0.2741	0.8719
14	45,X/46,X+mar(Y)	4.4/4.7	0.5248	2.14/2.04	0.7435	0.6895
15	45,X/46,XX	3.75/4.05	0.4647	1.80/2.06	4.399	0.1108
16	45,X/46,X,+mar(X)	4/4.3	0.5228	1.90/1.90	0.1673	0.9198
17	45,X/46,X,i(Yp)	4.35/3.3	0.0703	1.96/2.06	1.000	0.6065

* *p* ≤ 0.05, ** *p* ≤ 0.01. SCE = sister chromatid exchange, PI = proliferation index, χ^2^ = chi-squared test.

## Data Availability

The data presented in this study are available by the corresponding author upon request.

## Supplementary Materials

The following supporting information can be downloaded at: https://www.mdpi.com/article/10.3390/life14070848/s1, Table S1: phenotypes and other clinical characteristics of mosaic TS participants included in the present study. Figure S1: participant 1 karyotype analysis; FISH technique of interphase nuclei using specific centromeric X chromosome probe DXZ1. Figure S2: participant 2 karyotype analysis; FISH technique of interphase nuclei using specific centromeric X chromosome probe DXZ1. Figure S3: participant 3 karyotype analysis; FISH technique of interphase nucleus using specific centromeric X chromosome probe DXZ1, partial metaphases using specific centromeric X chromosome probe DXZ1, and partial metaphases using LSI XIST probe. Figure S4: participant 4 karyotype analysis; FISH technique of partial metaphases using whole-chromosome paint (WCP) probe for Y chromosome, partial metaphases using specific centromeric X chromosome DXZ1 probe and specific centromeric Y chromosome probe DYZ3, partial metaphases using Locus Specific Identifier (LSI) SHOX probe, partial metaphases using LSI SRY probe. Figure S5: participant 5 karyotype analysis; FISH technique of interphase nucleus using specific centromeric X chromosome probe DXZ1, partial metaphases using specific centromeric X chromosome probe DXZ1, partial metaphases using Locus Specific Identifier (LSI) XIST probe. Figure S6: participant 6 karyotype analysis; FISH technique of partial metaphases using specific centromeric X chromosome probe DXZ1, partial metaphases using Locus Specific Identifier (LSI) XIST probe. Figure S7: participant 7 karyotype analysis; FISH technique of interphase nuclei using specific centromeric X chromosome probe DXZ1, interphase nuclei using Locus Specific Identifier (LSI) XIST probe. Figure S8: participant 8 karyotype analysis; FISH technique of partial metaphases using specific centromeric X chromosome probe DXZ1, partial metaphases using Locus Specific Identifier (LSI) XIST probe. Figure S9: participant 9 karyotype analysis; FISH technique of interphase nucleus using specific centromeric X chromosome probe DXZ1, interphase nucleus using Locus Specific Identifier (LSI) SRY probe. Figure S10: participant 10 karyotype analysis; FISH technique of interphase nucleus using specific centromeric X chromosome probe DXZ1, partial metaphases using specific centromeric X chromosome probe DXZ1, partial metaphases using Locus Specific Identifier (LSI) XIST probe. Figure S11: participant 11 karyotype analysis; FISH technique of partial metaphases using specific centromeric X chromosome probe DXZ1, partial metaphases using Locus Specific Identifier (LSI) XIST probe. Figure S12: participant 12 karyotype analysis; FISH technique of interphase nucleus using specific centromeric X chromosome probe DXZ1, partial metaphases using specific centromeric X chromosome probe DXZ1, partial metaphases using Locus Specific Identifier (LSI) XIST probe. Figure S13: participant 13 karyotype analysis; FISH technique of partial metaphases using specific pericentromeric X and Y chromosome probe ENXY, partial metaphases using Locus Specific Identifier (LSI) XIST probe. Figure S14: participant 14 karyotype analysis; FISH technique of partial metaphases using specific pericentromeric X and Y chromosome probe ENXY, partial metaphases using specific centromeric Y chromosome probe DYZ3 and specific centromeric X chromosome probe DXZ1, partial metaphases using Locus Specific Identifier (LSI) SRY probe. Figure S15: participant 15 karyotype analysis; FISH technique of interphase nucleus using specific pericentromeric X and Y chromosome probe ENXY. Figure S16: participant 16 karyotype analysis; FISH technique of interphase nucleus using specific pericentromeric X and Y chromosome probe ENXY, partial metaphases using specific centromeric X chromosome probe DXZ1, partial metaphases using Locus Specific Identifier (LSI) XIST probe. Figure S17: participant 17 karyotype analysis; FISH technique of partial metaphases using specific pericentromeric X and Y chromosome ENXY probe, partial metaphases using specific centromeric Y chromosome probe DYZ3 and specific centromeric X chromosome probe DXZ1, partial metaphases using Locus specific Identifier (LSI) SHOX probe, partial metaphases using LSI SRY probe. Figure S18: Mosaic TS patients’ sexual and marker chromosome catalog. Figure S19: Frequencies of 45,X and 46,XN cell lineages after different incubation times. Figure S20: Micrographs of metaphases in the second division of participants 1 to 6 displaying sister chromatid exchanges. Figure S21: Micrographs of metaphases in the second division of participants 7 to 12 displaying sister chromatid exchanges. Figure S22: Micrographs of metaphases in the second division of participants 13 to 17 displaying sister chromatid exchanges.
