# Supplementary material for: Cell Cycle Kinetics and Sister Chromatid Exchange in Mosaic Turner Syndrome
Source: Life (Basel). 2024 Jul 5;14(7):848. doi: 10.3390/life14070848 (PMC11278208; doi:10.3390/life14070848)
Supplement: Supplementary file 1 [file life-14-00848-s001.zip › Supplemental figure S18.docx]

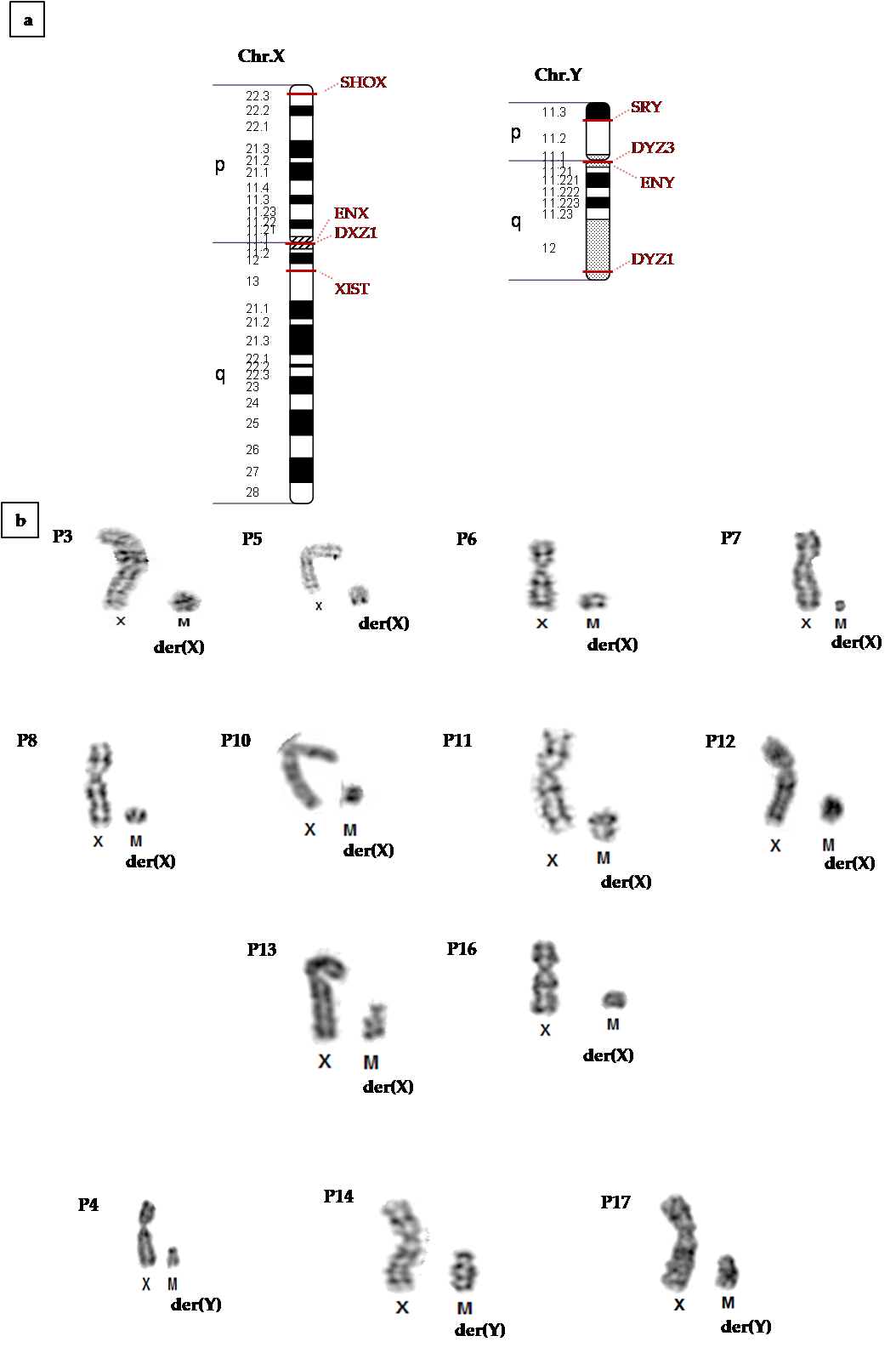


**Figure S18.** (a) Loci on X and Y chromosomes used to characterize anomalous chromosomes by FISH analyses; (b) characterization of anomalous chromosomes derived from X and Y chromosomes: anomalous chromosomes of participants 3, 5-8, 10-13, and 16 derived from X chromosome and anomalous chromosomes of participants 4, 14, and 17 derived from Y chromosome.
