# Supplementary material for: Cell Cycle Kinetics and Sister Chromatid Exchange in Mosaic Turner Syndrome
Source: Life (Basel). 2024 Jul 5;14(7):848. doi: 10.3390/life14070848 (PMC11278208; doi:10.3390/life14070848)
Supplement: Supplementary file 1 [file life-14-00848-s001.zip › Supplemental figures_S1-S17.docx]

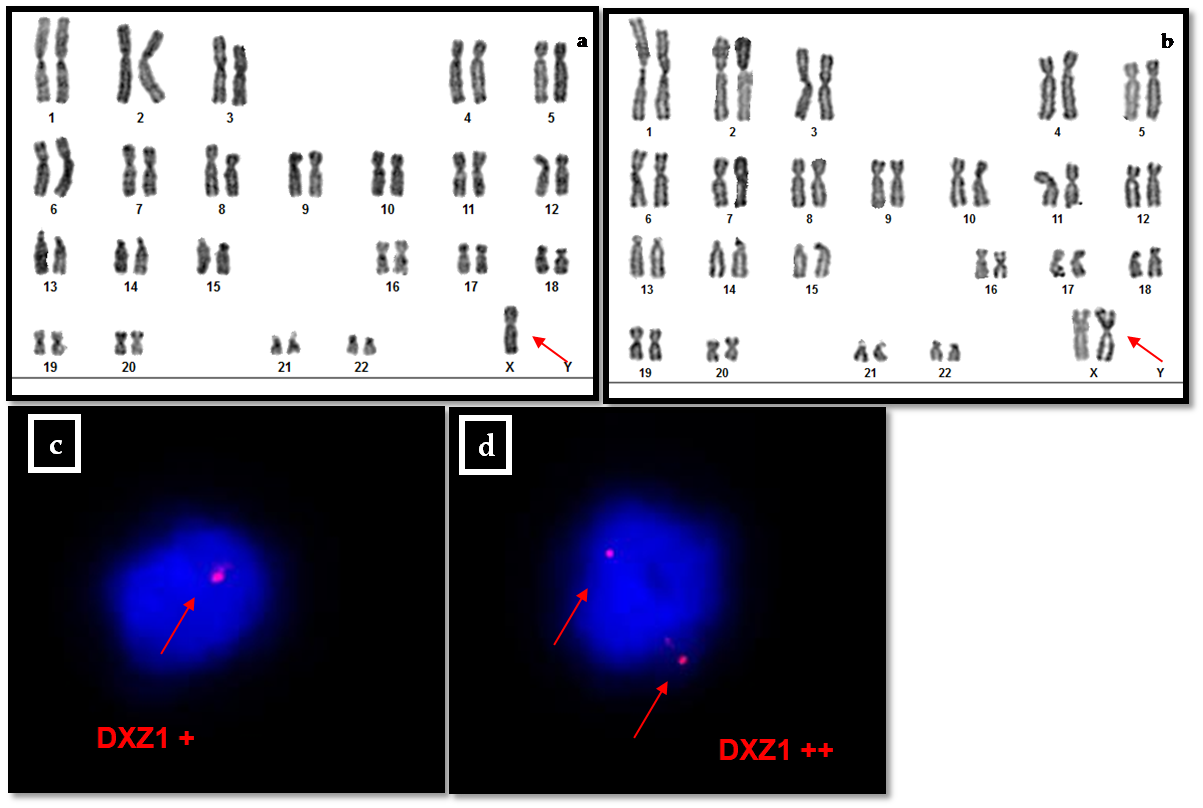


**Figure S1.** Participant 1 karyotype analysis: a) GTG-banded karyotype showing the 45,X cell lineage and b) GTG-banded karyotype showing the 46,XX cell lineage; X chromosomes indicated by red arrows. Interphase nuclei stained with specific centromeric X chromosome probe DXZ1 in FISH technique showing: c) one signal of DXZ1 (red) on 45,X cell and d) two signals of DXZ1 (red) on 46,XX cell indicated by red arrows.

Karyotype: mos45,X[54]/46,XX[46]

**
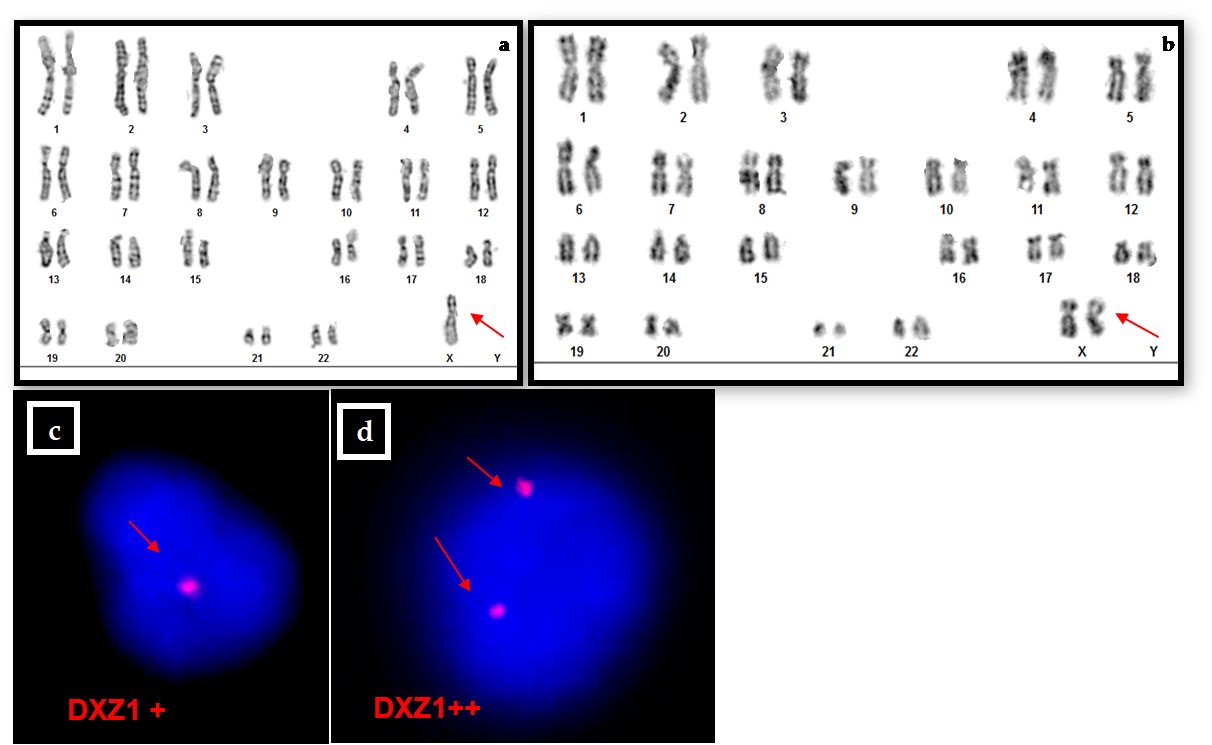
**

**Figure S2.** Participant 2 karyotype analysis: a) GTG-banded karyotype showing the 45,X cell lineage and b) GTG-banded karyotype showing the 46, XX cell lineage; X chromosomes indicated by red arrows. Interphase nuclei stained with specific centromeric X chromosome probe DXZ1 in FISH technique showing: c) one signal of DXZ1 (red) on 45,X cell and d) two signals of DXZ1 (red) on 46,XX cell indicated by red arrows.

Karyotype: mos45,X[58]/46,XX[42]

**
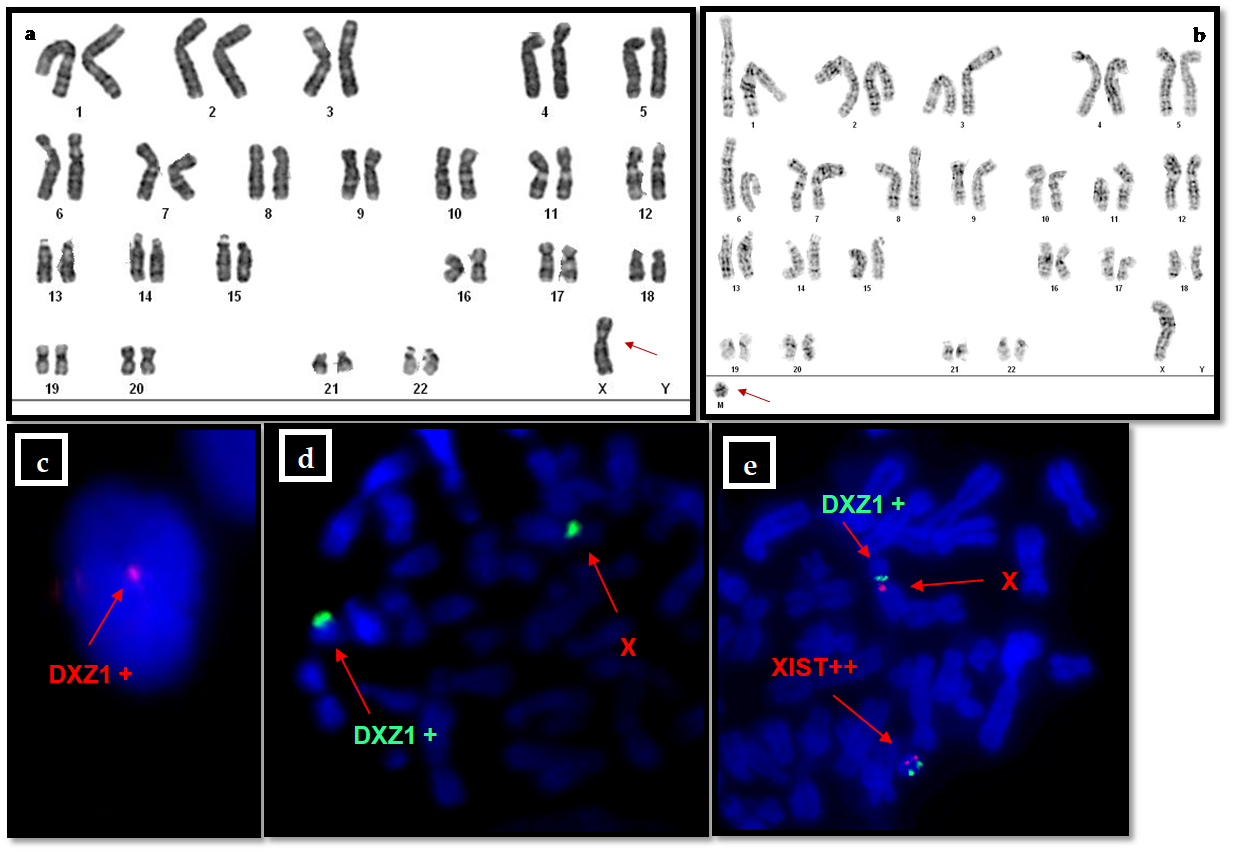
**

**Figure S3.** Participant 3 karyotype analysis: a) GTG-banded karyotype showing the 45,X cell lineage and b) GTG-banded karyotype showing a small ring chromosome indicated by a red arrow. FISH technique: c) interphase nucleus stained with specific centromeric X chromosome probe DXZ1 showing one signal of DXZ1 (red) on 45,X cell indicated by red arrow; d) partial metaphases stained with specific centromeric X chromosome probe DXZ1 showing one signal of DXZ1 (green) on normal size X chromosome and a split signal on small ring chromosome derived from X chromosome, both indicated by red arrows; e) partial metaphases stained with Locus Specific Identifier (LSI) XIST probe showing one signal of XIST (red) on normal size X chromosome and two signals on small ring chromosome, besides one centromeric X chromosome probe DXZ1 signal (green) on normal size X chromosome and two signals on small ring chromosome, confirming that it is a dicentric ring chromosome; both probe signals are indicated by red arrows.

Karyotype: mos45,X[76]/46,X,+r[24].ish r(X)(DXZ1+,*XIST*++)

**Figure S4.** Participant 4 karyotype analysis: a) GTG-banded karyotype showing the 45,X cell lineage and b) GTG-banded karyotype showing a small marker chromosome indicated by a red arrow. FISH technique: c) partial metaphases stained with specific centromeric X chromosome probe DXZ1 showing one signal of DXZ1 (green) on normal size X chromosome (in a 45,X cell lineage) indicated by a red arrow; d) partial metaphases stained with whole chromosome paint (WCP) probe for Y chromosome showing a marker chromosome derived from Y chromosome (red) indicated by a red arrow; e) partial metaphases stained with specific centromeric Y chromosome probe showing one signal of DXZ1 (green) on normal size X chromosome and two signals of DYZ3 (red) on marker chromosome; f) partial metaphases stained with Locus Specific Identifier (LSI) SHOX probe showing one signal on normal size X chromosome and two signals on marker chromosome, indicated by red arrows; g) partial metaphases stained with DXZ1 and LSI SRY probes showing one signal of DXZ1 (green) on normal size X chromosome and two signals of SRY on marker chromosome, both indicated by red arrows.

Karyotype: mos45,X[52]/46,X+mar[48].ish der(Y)(wcpY+,DYZ3++,*SHOX*++,*SRY*++)

**
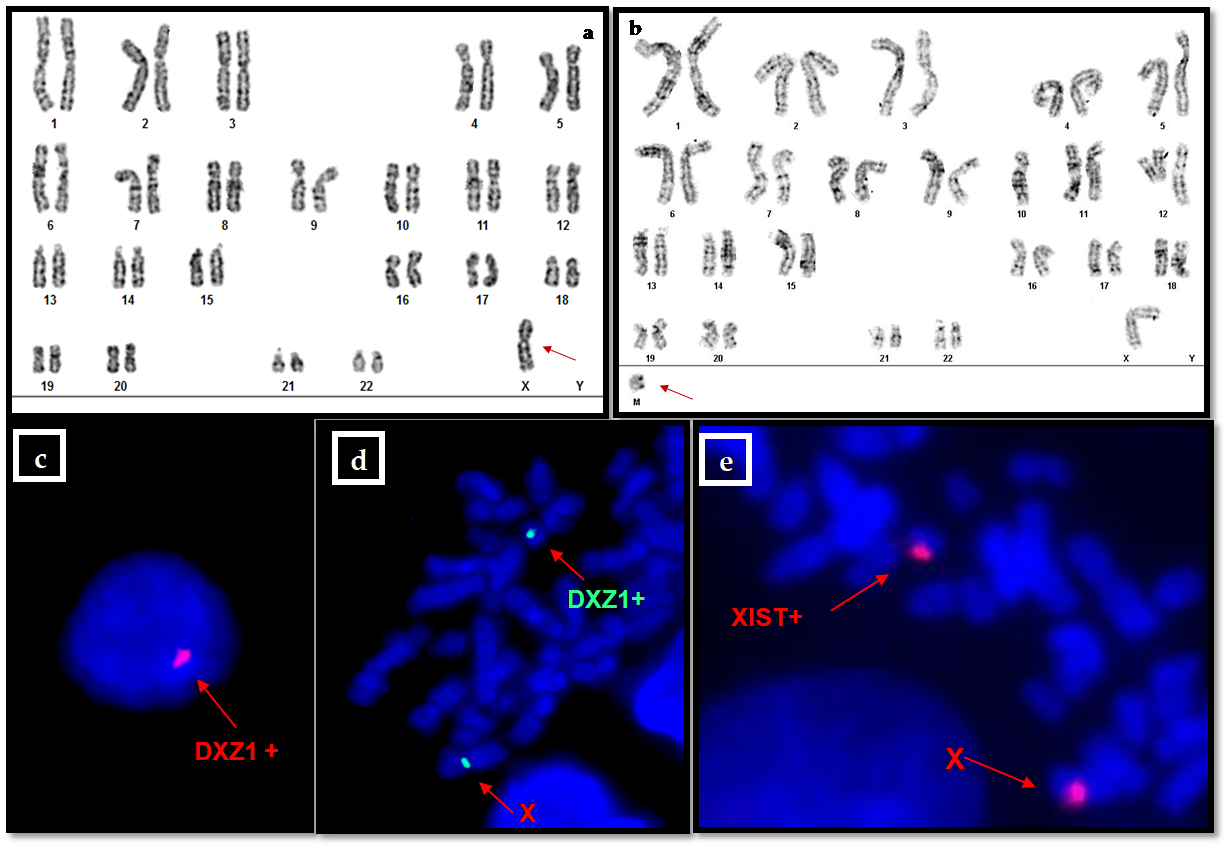
**

**Figure S5.** Participant 5 karyotype analysis: a) GTG-banded karyotype showing the 45,X cell lineage and b) GTG-banded karyotype showing a marker chromosome indicated by a red arrow. FISH technique of interphase nucleus stained using specific centromeric X chromosome probe DXZ1 showing: c) one signal of DXZ1 (red) on 45,X cell indicated by a red arrow. FISH technique: d) partial metaphases stained with centromeric X chromosome probe DXZ1 showing one signal of DXZ1 (green) on normal size X chromosome and one signal on marker chromosome derived from X chromosome, both indicated by red arrows; e) partial metaphases stained with Locus Specific Identifier (LSI) XIST probe showing one signal of XIST (red) on normal size X chromosome and on marker chromosome indicated by red arrows.

Karyotype: mos45,X[58]/46,X,+mar[42].ish der(X)(DXZ1+,*XIST*+)

**
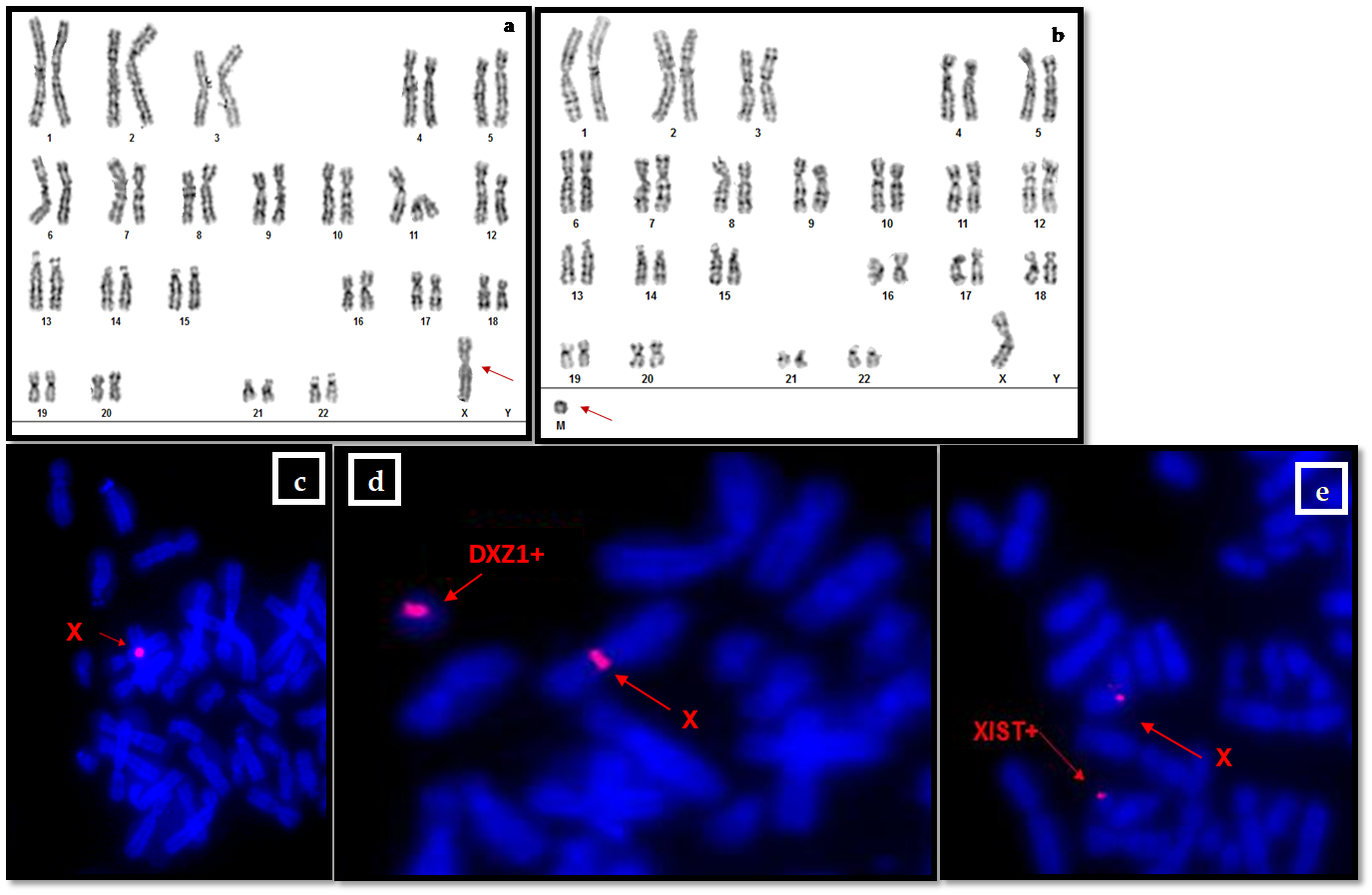
**

**Figure S6**. Participant 6 karyotype analysis: a) GTG-banded karyotype showing the 45,X cell line and b) GTG-banded karyotype showing the marker chromosome indicated by a red arrow. FISH technique: c) partial metaphases stained with specific centromeric X chromosome probe DXZ1 showing one signal of DXZ1 (red) on normal size X chromosome (in a 45,X cell lineage) indicated by red arrow; d) one signal of DXZ1 (red) on normal size X chromosome and one signal on marker chromosome derived from X chromosome indicated by red arrows; e) partial metaphases stained with Locus Specific Identifier (LSI) XIST probe showing one signal of XIST (red) on normal size chromosome and on marker chromosome (red) indicated by red arrows.

Karyotype: mos46,X,+mar[51]/45,X[49].ish der(X)(DXZ1+, *XIST*+)


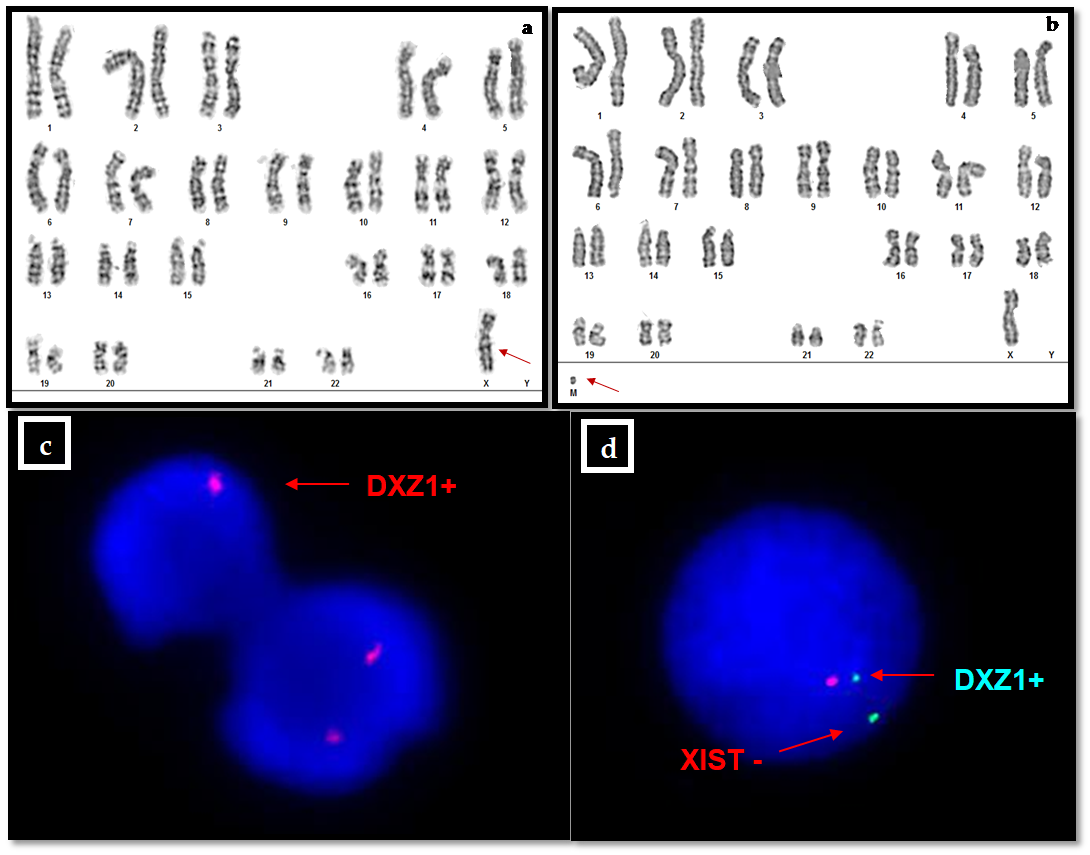


**Figure S7.** Participant 7 karyotype analysis: a) GTG-banded karyotype showing the 45,X cell line and b) GTG-banded karyotype showing the marker chromosome indicated by a red red arrow. FISH technique: c) interphase nuclei stained using specific centromeric X chromosome probe DXZ1 (red) showing one signal of DXZ1 in 45,X cell and two signals in 46,X+mar cell indicated by red arrows; d) interphase nuclei stained using Locus Specific Identifier (LSI) XIST probe (red) showing XIST negative marker chromosome and XIST positive normal size X chromosome accompanied by one centromeric X chromosome probe DXZ1 signal (green) on normal size X chromosome and marker chromosome, indicated by red arrows.

Karyotype: mos45,X[94]/ 46,X,+mar[6].ish der(X)(DXZ1+,*XIST*-)


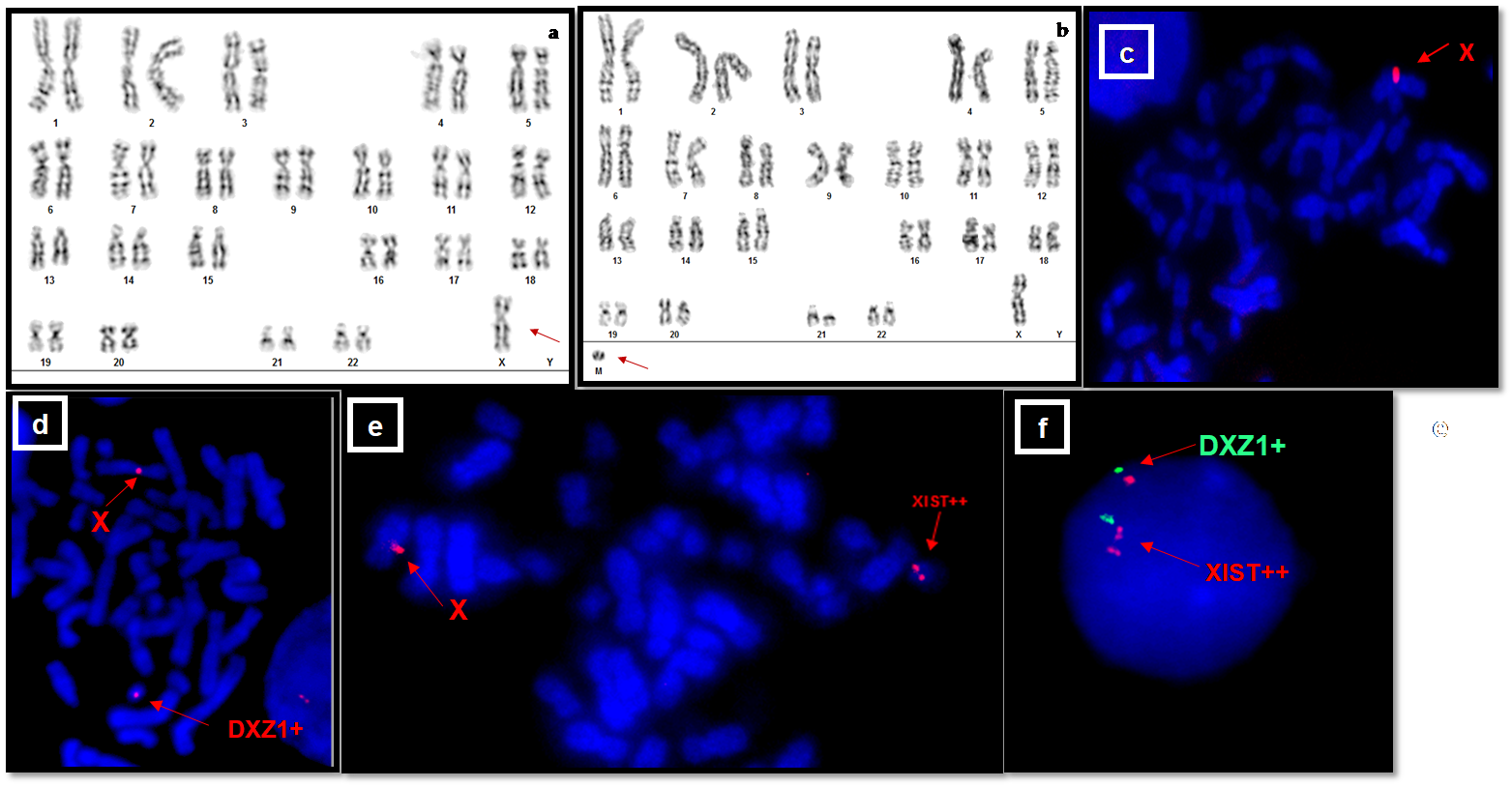


**Figure S8.** Participant 8 karyotype analysis: a) GTG-banded karyotype showing the 45,X cell line and b) GTG-banded karyotype showing the marker chromosome indicated by a red arrow. FISH technique: c) partial metaphases stained with specific centromeric X chromosome probe DXZ1 showing one signal of DXZ1 (red) on normal size X chromosome (in a 45,X cell lineage) and d) one signal of DXZ1 (red) on normal size X chromosome and one signal on marker chromosome derived from X chromosome, both indicated by red arrows; e) partial metaphases using Locus Specific Identifier (LSI) XIST probe showing two signals of XIST (red) on marker chromosome indicated by a red arrow; f) interphase nucleus using Locus Specific Identifier (LSI) XIST (red) probe having an internal chromosomal centromeric control, DXZ1 (green), showing one signal of XIST (red) on normal size X chromosome and two signals on marker chromosome, along with one signal of the centromeric control, DXZ1 (green), on both chromosomes in a 46,X +mar cell, all indicated by red arrows.

Karyotype: mos46,X,+mar[58]/45,X[42].ish der(X)(DXZ1+,*XIST++*)

**Figure S9.** Participant 9 karyotype analysis: a) GTG-banded karyotype showing the 45,X cell lineage and b) GTG-banded karyotype showing 46,XY cell lineage indicated by red arrows. FISH technique: c) interphase nucleus stained with Locus Specific Identifier( LSI) SRY probe showing one signal of DXZ1 (green) on 45,X cell, and d) one signal of DXZ1 (green) and one signal of SRY on 46,XY cell indicated both by red arrows.

Karyotype: mos46,XY[53]/45,X[47]


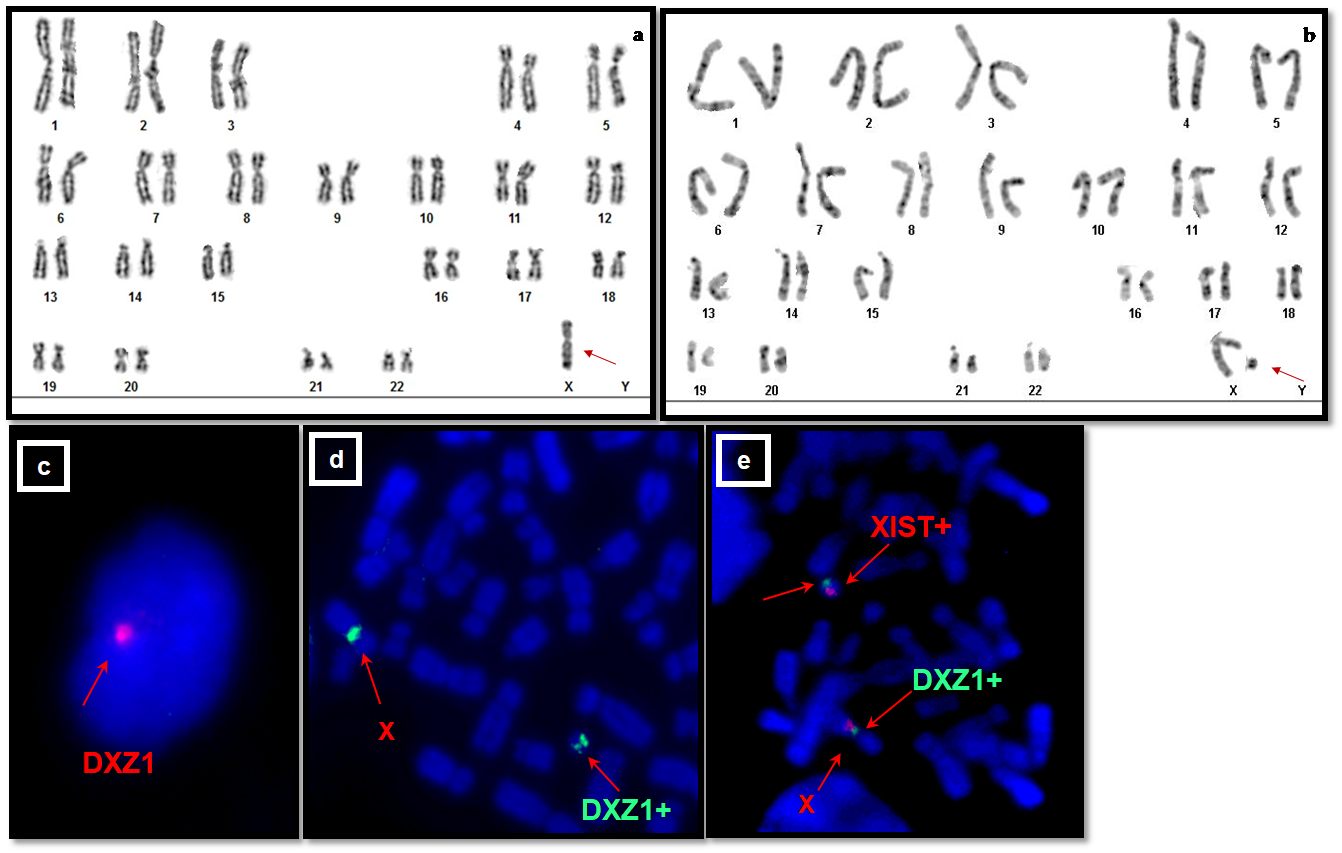


**Figure S10.** Participant 10 karyotype analysis: a) GTG-banded karyotype showing the 45,X cell line and b) GTG-banded karyotype showing the marker chromosome indicated by a red arrow. FISH technique: c) interphase nucleus using specific centromeric X chromosome probe DXZ1 showing one signal of DXZ1 (red) in a 45,X cell indicated by a red arrow; d) partial metaphases using specific centromeric X chromosome probe DXZ1 showing one signal of DXZ1 (green) on normal size X chromosome and one signal on marker chromosome derived from X chromosome, both indicated by red arrows; e) partial metaphases using Locus Specific Identifier (LSI) XIST probe having an internal chromosomal centromeric control, DXZ1 (green), showing one signal of XIST (red) and one signal of DXZ1 (green) on normal size X chromosome and on marker chromosome, both indicated by red arrows.

Karyotype: mos46,X,+mar[58]/45,X[42].ish der(X)(DXZ1+,*XIST+*)


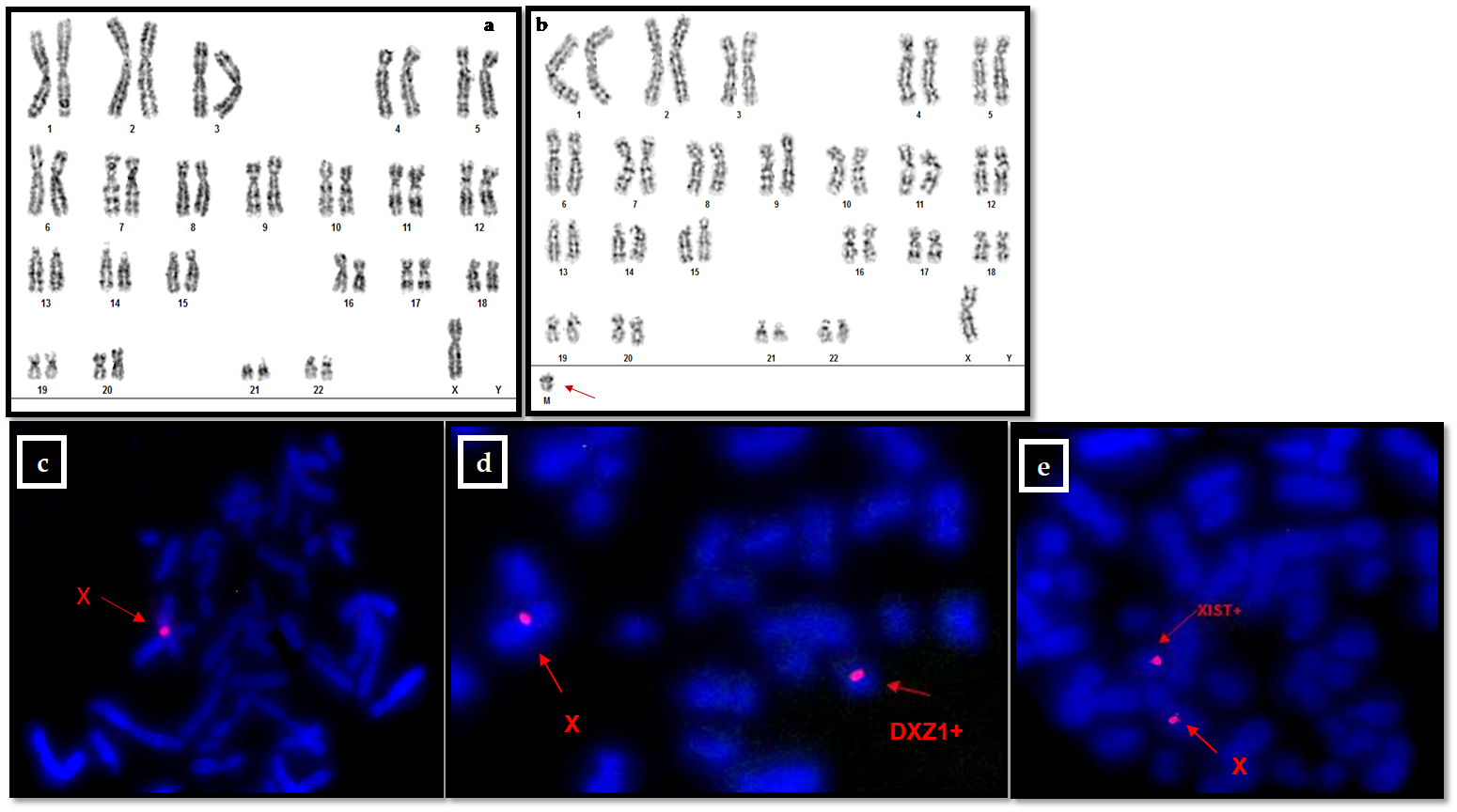


**Figure S11.** Participant 11 karyotype analysis: a) GTG-banded karyotype showing the 45,X cell line and b) GTG-banded karyotype showing the marker chromosome indicated by a red arrow. FISH technique: c) partial metaphases stained with specific centromeric X chromosome probe DXZ1 showing one signal of DXZ1(red) on normal size X chromosome (in a 45,X cell lineage) indicated a by red arrow, and d) one signal of DXZ1 (red) on normal size X chromosome and one signal on marker chromosome derived from X chromosome, both indicated by red arrows; e) partial metaphases stained with Locus Specific Identifier (LSI) XIST probe showing one signal of XIST (red) on normal size X chromosome and on marker chromosome, both indicated by red arrows.

Karyotype: mos45,X[51]/46,X,+mar[49].ish der(X)(DXZ1+,*XIST*+)


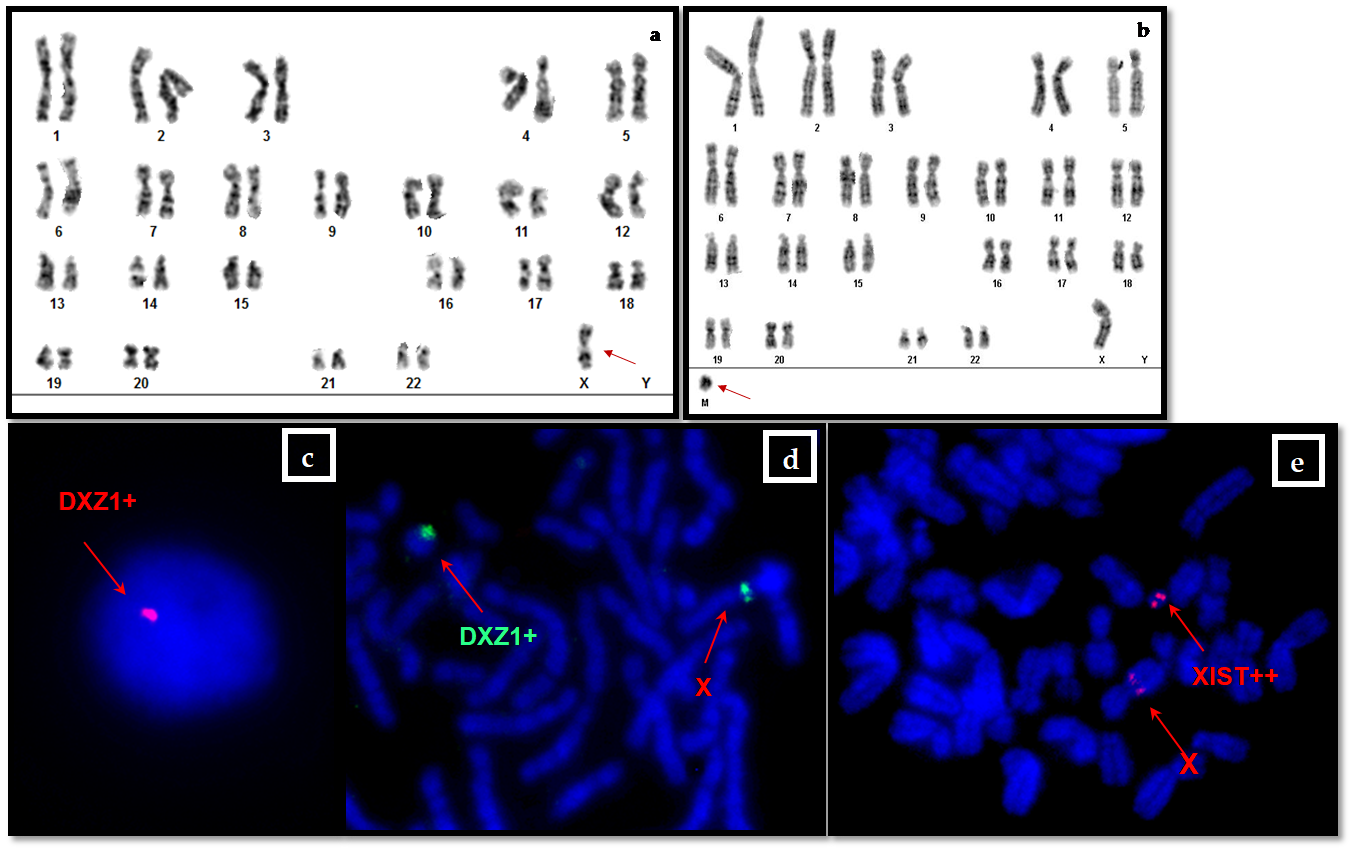


**Figure S12.** Participant 12 karyotype analysis: a) GTG-banded karyotype showing the 45,X cell line and b) GTG-banded karyotype showing the marker chromosome indicated by a red arrow. FISH technique: c) interphase cell stained with specific centromeric X chromosome probe DXZ1 showing one signal of DXZ1 (red) in a 45,X cell indicated by a red arrow; d) partial metaphases stained with specific centromeric X chromosome probe DXZ1 showing one signal of DXZ1 (green) on normal size X chromosome and one signal on marker chromosome derived from X chromosome, both indicated by red arrows; e) partial metaphases stained with Locus Specific Identifier (LSI) XIST probe showing one signal of XIST (red) on normal size X chromosome and two signals on marker chromosome indicated by red arrows.

Karyotype: mos45,X[61]/ 46,X,+mar[39].ish der(X)(DXZ1+,*XIST*++)


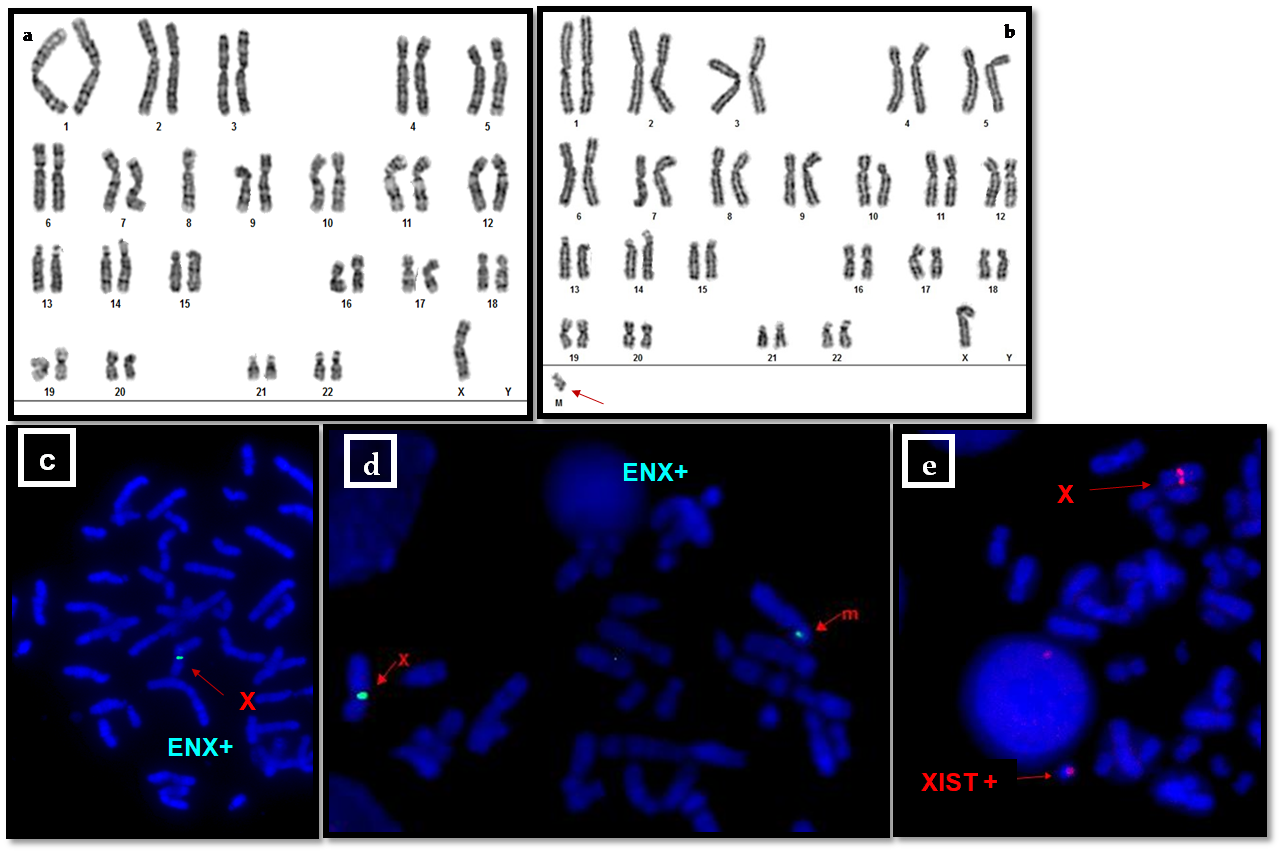


**Figure S13.** Participant 13 karyotype analysis:  a) GTG-banded karyotype showing the 45,X cell line and  b) GTG-banded karyotype showing the marker chromosome indicated by a red arrow. FISH technique: c) partial metaphases stained with specific pericentromeric X and Y chromosome ENXY probe showing one signal of ENX (green) on normal size X chromosome (in a 45,X line) indicated by red arrow, and d) one signal of ENX (green) on normal size X chromosome and one signal on marker chromosome derived from X chromosome, both indicated by red arrows; e) partial metaphases stained with Locus Specific Identifier (LSI) XIST probe showing one signal of XIST (red) on normal size X chromosome and on marker chromosome, both indicated by red arrows.

Karyotype: mos45,X[82]/ 46,X,+mar[18].ish der(X)(ENX+,*XIST*+)

**Figure S14.** Participant 14 karyotype analysis:  a) GTG-banded karyotype showing the 45,X cell line and  b) GTG-banded karyotype showing the marker chromosome indicated by a red arrow. FISH technique: c) partial metaphases stained with specific pericentromeric X and Y chromosome probe ENXY showing one signal of ENX on normal size X chromosome (green) (in a 45,X cell lineage) indicated by red arrow and d) one signal of ENX on normal size X chromosome (green) and two signals of ENY (red) on isodicentric marker chromosome derived from Y chromosome, both indicated by red arrows; e) partial metaphases stained with specific centromeric Y chromosome probe showing one signal of DXZ1 (green) on normal size X chromosome and two signals of DYZ3 (red) on marker chromosome; f) partial metaphases stained with Locus Specific Identifier (LSI) SRY probe showing one signal of DXZ1 (green) on normal size X chromosome and two signals of SRY (red) on marker chromosome, both indicated by red arrows.

Karyotype: mos46,X,+mar[55]/45,X[45].ish der(Y)(ENY++,DYZ3++,*SRY++*)

**
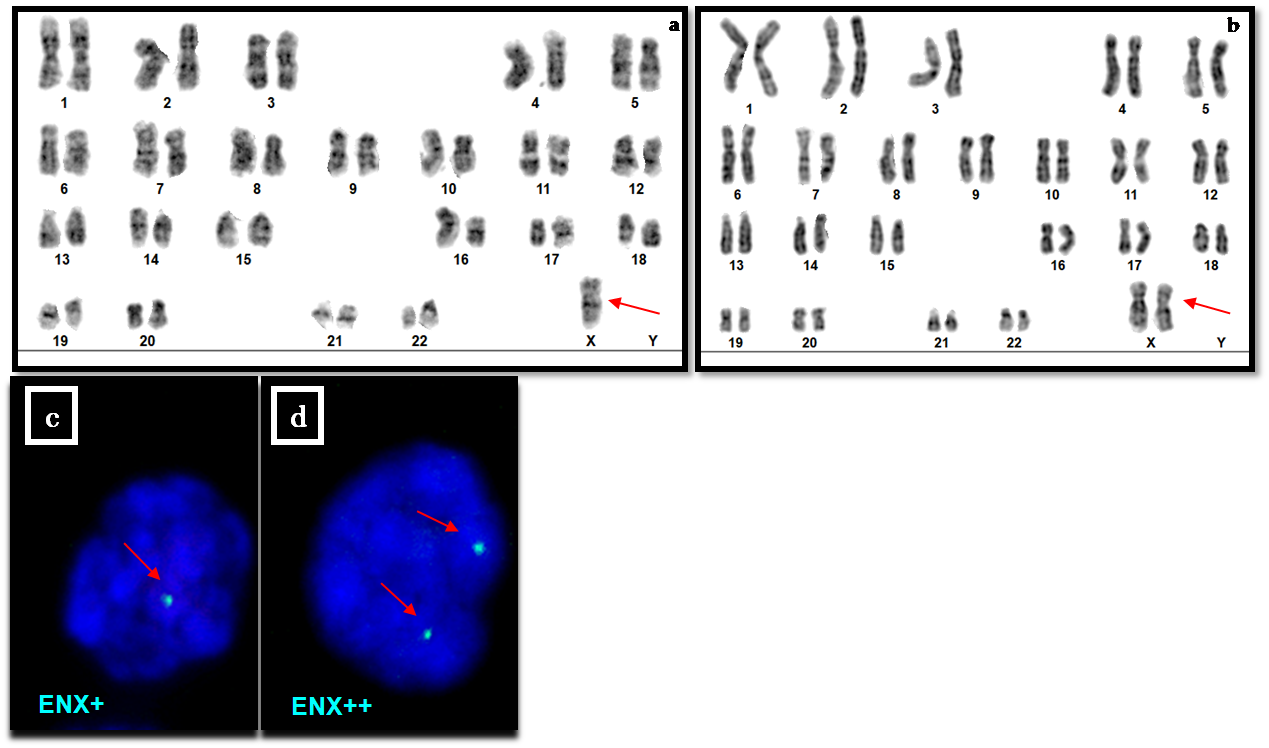
**

**Figure S15.** Participant 15 karyotype analysis: a) GTG-banded karyotype showing the 45,X cell lineage and b) GTG-banded karyotype showing the 46,XX cell lineage, both indicated by red arrows. FISH technique: c) interphase nucleus stained with specific pericentromeric X and Y chromosome probe ENXY showing one signal of ENX (green) on 45,X cell and d) two signals of ENX (green) on 46,XX cell, both indicated by red arrows.

Karyotype: mos45,X[57]/46,XX[43]

**Figure S16.** Participant 16 karyotype analysis: a) GTG-banded karyotype showing the 45,X cell line and b) GTG-banded karyotype showing the marker chromosome indicated by a red arrow. FISH technique: c) interphase nucleus stained with specific pericentromeric X and Y chromosome probe ENXY showing one signal of ENX in a 45,X cell (green) indicated by a red arrow; d) partial metaphases stained with specific centromeric X chromosome probe DXZ1 showing one signal of DXZ1 (red) on normal size X chromosome and one signal on marker chromosome derived from X chromosome, both indicated by yellow arrows; e) partial metaphases stained with Locus Specific Identifier (LSI) XIST probe showing one signal of XIST (red) on normal size X chromosome and on marker chromosome, both indicated by red arrows.

Karyotype: mos45,X[53]/ 46,X,+mar[47].ish der(X)(DXZ1+, *XIST*+)


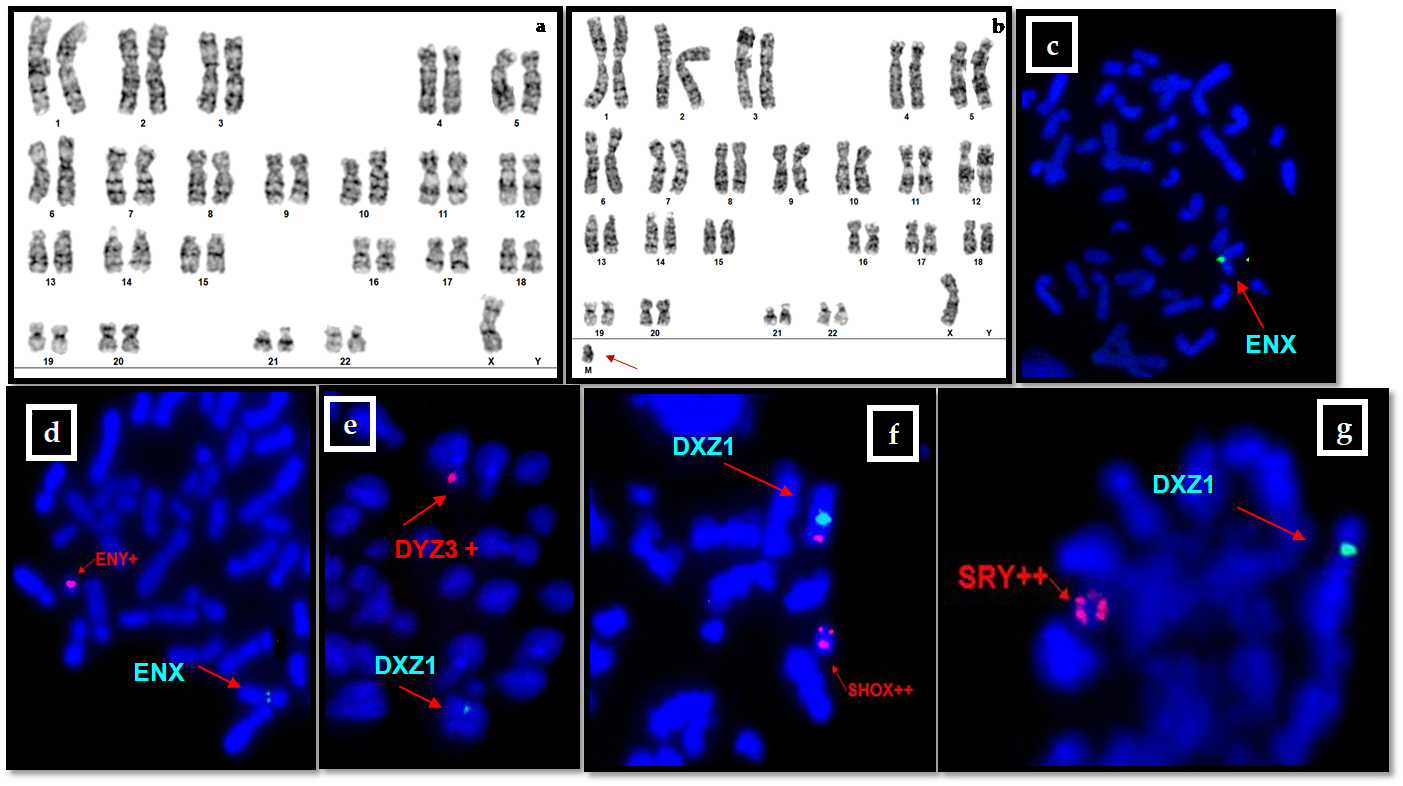


**Figure S17.** Participant 17 karyotype analysis: a) GTG-banded karyotype showing the 45,X cell line and b) GTG-banded karyotype showing an isochromosome for short arm of Y chromosome indicated by a red arrow. FISH technique: c) partial metaphases stained with specific pericentromeric X and Y chromosome ENXY probe showing one signal of ENX on normal size X chromosome (green) (in a 45,X cell lineage) and d) one signal of ENX on X normal chromosome (green) and one signal of ENY (red) on isochromosome for short arm of Y chromosome, both indicated by red arrows; e) partial metaphases stained with specific centromeric Y chromosome probe DYZ3 showing one signal of DXZ1 on X normal chromosome and one signal of DYZ3 on isochromosome for short arm of Y chromosome; f) partial metaphases stained with Locus Specific Identifier (LSI) SHOX probe showing one signal of DXZ1 (green) and one signal of SHOX (red) on normal X chromosome and two signals on isochromosome for short arm of Y chromosome; g) partial metaphases stained with LSI SRY probe showing one signal of DXZ1 (green) on normal X chromosome and two signals of SRY (red) on isochromosome for short arm of Y chromosome, both indicated by red arrows.

Karyotype: mos46,X,i(Y)(p10)[81]/45,X[19].ishi(Y)(ENY+,DYZ3+,*SRY++*,*SHOX++*)
