# Supplementary material for: Cell Cycle Kinetics and Sister Chromatid Exchange in Mosaic Turner Syndrome
Source: Life (Basel). 2024 Jul 5;14(7):848. doi: 10.3390/life14070848 (PMC11278208; doi:10.3390/life14070848)
Supplement: Supplementary file 1 [file life-14-00848-s001.zip › Supplemental figures_S20-S22.docx]

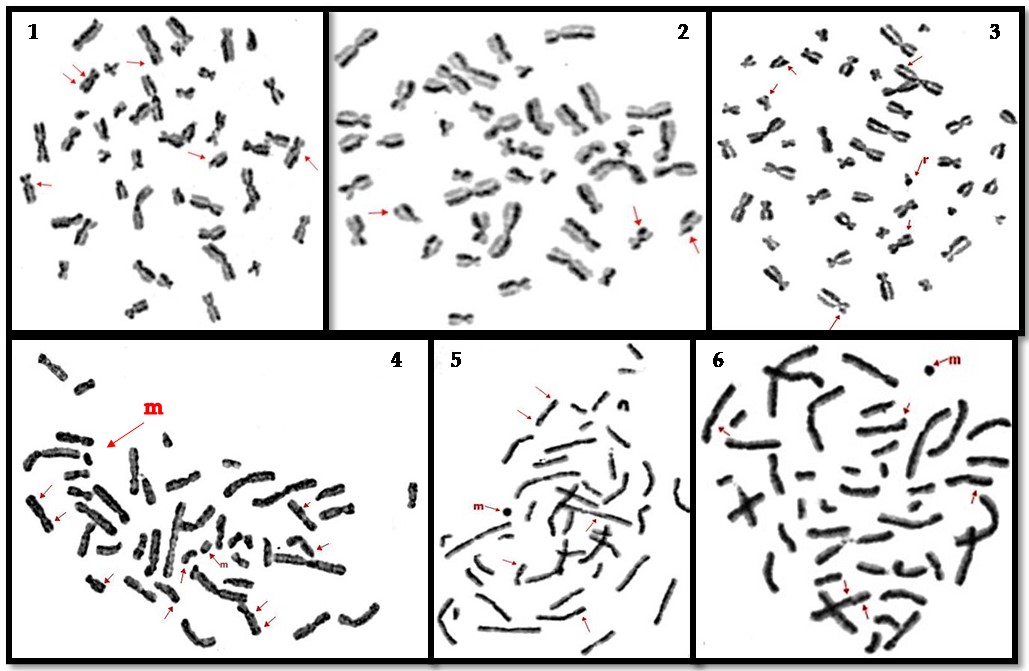


**Figure S20.** Micrographs of metaphases in second division of participants 1 to 6 displaying sister chromatid exchanges (red arrows).

m=marker chromosome (sSMC) and r=ring chromosome (red arrows).


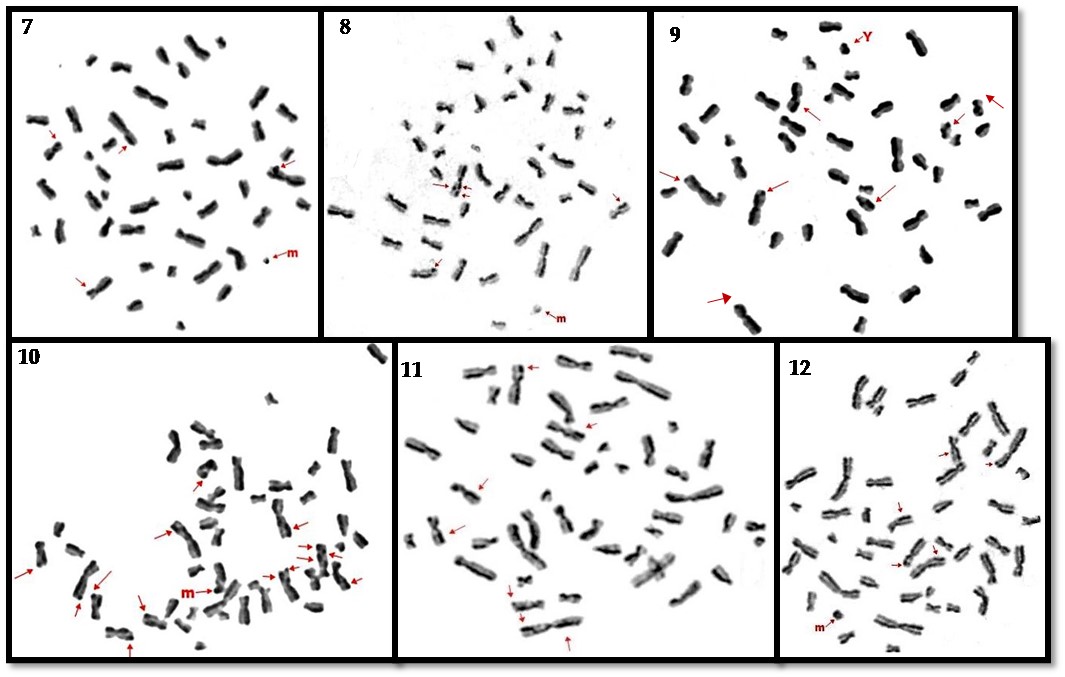


**Figure S21.** Micrographs of metaphases in second division of participants 7 to 12 displaying sister chromatid exchanges (red arrows).

m=marker chromosome (sSMC) and Y =Y chromosome (red arrows).


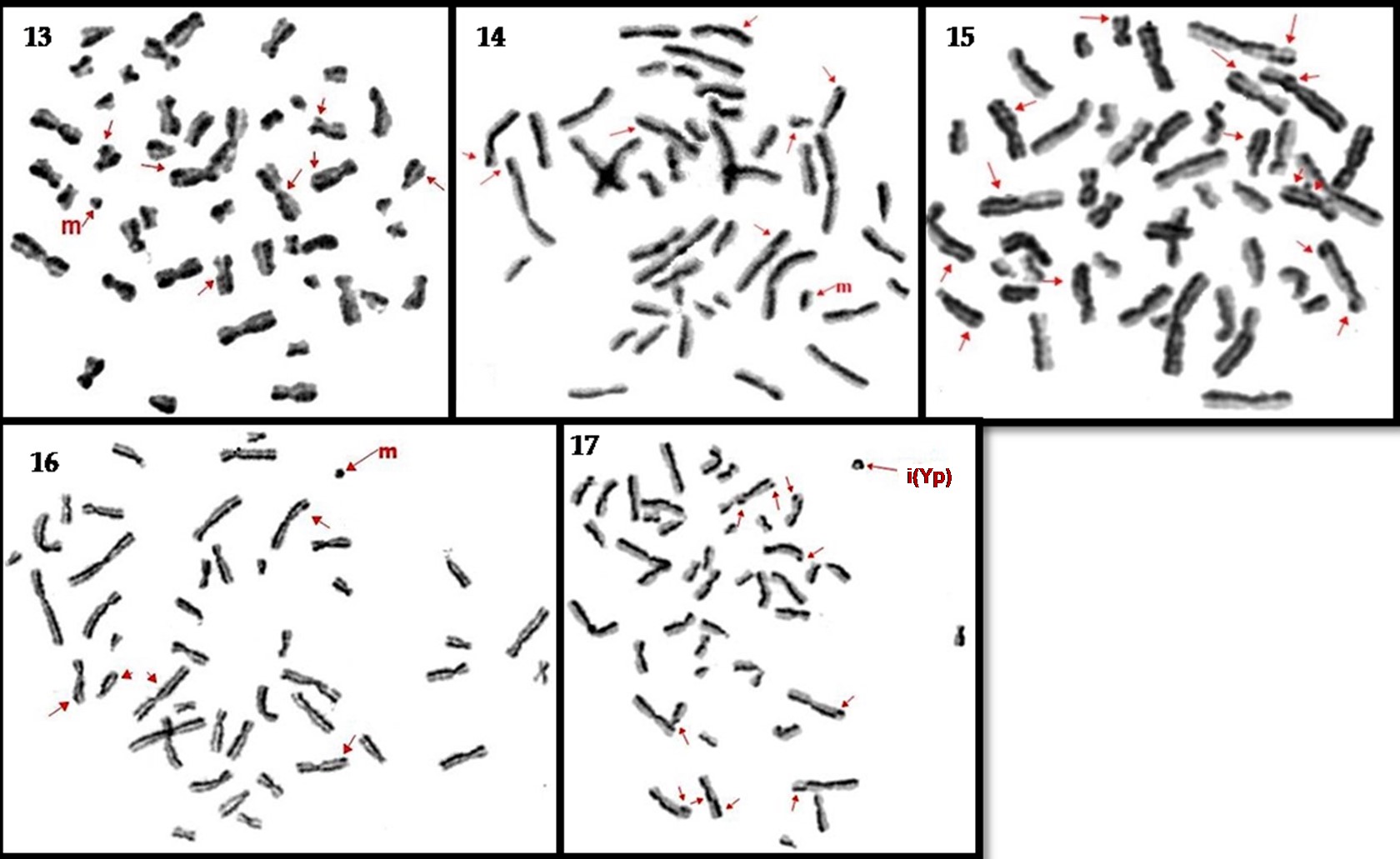


**Figure S22.** Micrographs of metaphases in second division of participants 13 to 17 displaying sister chromatid exchanges (red arrows).

m=marker chromosome (sSMC) and i(Yp) =isochromosome for short arm of the Y chromosome (red arrows).
