# Supplementary material for: Cell Cycle Kinetics and Sister Chromatid Exchange in Mosaic Turner Syndrome
Source: Life (Basel). 2024 Jul 5;14(7):848. doi: 10.3390/life14070848 (PMC11278208; doi:10.3390/life14070848)
Supplement: Supplementary file 1 [file life-14-00848-s001.zip › Table S1.docx]

| **Participant** | **Age at diagnosis**  **(years)** | **Clinical indication** | **Phenotype** |
| --- | --- | --- | --- |
| 1 | 14 | Short stature (SS) | SS, ocular hypertelorism, strabismus, telecanthus, ogival palate, prognathism, low set ears, *pterygium colli*, low posterior hairline, broad chest, *cubitus valgus*, multiple pigmented nevi (>10), lymphedema of hands and feet, hyperconvex nails, bicuspid aortic valve, small uterus, normal gonads, and spontaneous menarche. |
| 2 | 12 | Short stature (SS)  Amenorrhea | SS, ocular hypertelorism, ogival palate, *pterygium colli,* broad chest, sensorineural hearing loss, small uterus, non-visualized ovaries, induced menarche, 17 yr. at menarche, delayed puberty, primary amenorrhea, bone age delay, and osteopenia. |
| 3 | 7 | Short stature (SS) | SS, ocular hypertelorism, strabismus, ogival palate, prognathism, *pectus excavatum*, *cubitus valgus*, short fourth metacarpal, multiple pigmented nevi (>10), lymphedema of hands and feet, nails hypoplasia, small uterus, non-visualized ovaries, induced menarche, 17 yr. at menarche, delayed puberty, primary amenorrhea, subclinical hypothyroidism, and bone age delay. |
| 4 | 7 | Short stature (SS) | SS, ocular hypertelorism, ogival, palpebral ptosis, palate, low posterior hairline, triangular face, camptodactyly**,** multiple pigmented naevi (<10), small uterus, and prophylactic gonadectomy at 8 yr. |
| 5 | 12 | Short stature (SS) | SS, ocular hypertelorism, ogival palate, micrognathia, telecanthus, nail hypoplasia, small uterus, non-visualized ovaries, induced menarche, 15 yr. at menarche, delayed puberty, primary amenorrhea, and bone age delay |
| 6 | 8 | Short stature (SS) | SS, ocular hypertelorism, ogival palate, telecanthus, *pectus excavatum*, c*ubitus valgus*, hyperconvex nails, multiple pigmented nevi (<10), small uterus, gonadal dysgenesis, delayed and induced puberty, and primary amenorrhea. |
| 7 | 1 | Short stature (SS) | SS, ocular hypertelorism, ogival palate, prognathism, *pterygium colli*, *pectus excavatum*, congenital anomalies of the kidneys, and prepubescent |
| 8 | 10 | Short stature (SS) | SS, synovial cyst, multiple pigmented nevi (<10), small uterus, gonadal dysgenesis, induced menarche, delayed puberty, primary amenorrhea, diabetes mellitus, and obesity. |
| 9 | 11 | Short stature (SS) | SS, ocular hypertelorism, ogival palate, short neck, broad chest, small uterus, prophylactic gonadectomy at 11 yr., and obesity. |
| 10 | 7 | Short stature (SS) and Horseshoe kidney | SS, ocular hypertelorism, ogival palate, low posterior hairline, strabismus, nails hypoplasia, multiple pigmented nevi (>10), horseshoe kidneys, small uterus, normal gonads, spontaneous menarche, 12 yr. at menarche, normal puberty, secondary amenorrhea, bone age delay, and obesity. |
| 11 | 3 | Short stature (SS) | SS, ogival palate, low posterior hairline, cubitus valgus, multiple pigmented naevi (<10), short fourth metacarpal, and prepubescent |
| 12 | 11 | Short stature (SS) | SS, ocular hypertelorism, strabismus, ogival palate, micrognathia, low posterior hairline, *pectus excavatum*, widely spaced nipples, *cubitus valgus*, short fourth metacarpal, bicuspid aortic valve, congenital anomalies of the kidneys and horseshoe kidney, small uterus, gonadal dysgenesis, induced menarche, 16 yr. at menarche, delayed puberty, primary amenorrhea, hypothyroidism, and obesity. |
| 13 | - | Short stature (SS) | Not informed |
| 14 | 15 | Short stature (SS)  Amenorrhea | SS, ogival palate, low posterior hairline, *cubitus valgus*, short fourth metacarpal, multiple pigmented nevi (<10), bicuspid aortic valve, small uterus, prophylactic gonadectomy at 15 yr., and obesity. |
| 15 | 14 | Short stature (SS) | SS, ogival palate, short neck, conductive hearing loss, nails hypoplasia, small uterus, non-visualized ovaries, spontaneous menarche, 15 yr. at menarche, delayed puberty, secondary amenorrhea, intellectual disability, bone age delay, and osteopenia. |
| 16 | 9 | Short stature (SS) | SS, ocular hypertelorism, upslanted palpebral fissures, ogival palate, prognathism, low posterior hairline, *cubitus valgus*, short fourth metacarpal, multiple pigmented naevi (>10), lymphedema of feet, nails hyperconvex, parapyelic cysts, small uterus, gonadal dysgenesis, spontaneous menarche, 14 yr. at menarche, delayed puberty, and secondary amenorrhea. |
| 17 | 14 | Short stature (SS) | SS, multiple pigmented nevi (<10), acanthosis nigricans**,** small uterus, prophylactic gonadectomy at 15 yr., and obesity. |
